# Associations of FGF23 and Apelin-13 with osteoporosis and diabetic peripheral neuropathy in postmenopausal women with type 2 diabetes mellitus

**DOI:** 10.3389/fmed.2026.1815912

**Published:** 2026-05-11

**Authors:** Yue Kong, Fan Zuo, Xuxiang Zhang, Zhao Xu, Yudi Hu, Xin Nian

**Affiliations:** Department of Endocrinology, The First Affiliated Hospital of Kunming Medical University, Kunming, China

**Keywords:** Apelin-13, FGF23, osteoporosis, peripheral neuropathy, postmenopausal, type 2 diabetes mellitus

## Abstract

**Objective:**

To investigate the correlations of various clinical indicators, fibroblast growth factor 23 (FGF23), and Apelin-13 with osteoporosis (OP) and diabetic peripheral neuropathy (DPN) in postmenopausal women with type 2 diabetes mellitus (T2DM), and to evaluate their predictive value for OP and DPN.

**Methods:**

A total of 320 postmenopausal women were enrolled, including 238 patients with T2DM and 82 non-diabetic patients with OP. Clinical data, bone metabolism markers, continuous glucose monitoring parameters, Current Perception Threshold (CPT), and Bone Mineral Density (BMD) were collected. Serum levels of FGF23 and Apelin-13 were measured via ELISA. Multivariate regression analyses identified independent factors for BMD, OP, and DPN in T2DM patients. Predictive performance was evaluated using ROC curves. The characteristic clinical phenotype of OP patients with comorbid T2DM was also summarized.

**Results:**

In postmenopausal women with T2DM, 25OH-VitD and Apelin-13 were independently associated with higher T-scores (protective factors), while PTH, CPT scores, and FGF23 were independently associated with lower T-scores (risk factors). Logistic regression revealed that longer DD, elevated superficial peroneal/saphenous nerve CPT scores, and higher FGF23 were independently associated with increased OP risk, while higher E2 and Apelin-13 were independently associated with decreased OP risk. The combined model of these indicators showed an AUC of 0.958 (95% CI: 0.934–0.982) for predicting OP. Logistic regression indicated that a lower BMD T-score was independently associated with increased DPN risk (OR = 0.13, *p* < 0.001), with a predictive AUC of 0.926 (95% CI: 0.891–0.961). OP patients with T2DM exhibited a more adverse metabolic-bone profile, including higher FGF23 and lower Apelin-13.

**Conclusion:**

In this cross-sectional study, decreased BMD in postmenopausal T2DM women was independently associated with bone metabolism disorders, FGF23/Apelin-13 imbalance, and impaired nerve function. OP and DPN shared long diabetes duration, high FGF23, and low Apelin-13. A model combining DD, E2, CPT, FGF23, and Apelin-13 showed good OP discrimination (AUC = 0.958); BMD T-score discriminated DPN (AUC = 0.926). Both require external validation. These hypothesis-generating findings suggest that FGF23 and Apelin-13 may be potential targets for future research on synergistic OP/DPN prevention and treatment.

## 1 Introduction

Type 2 diabetes mellitus (T2DM) significantly impairs quality of life and increases disability and mortality (1, 2). Its complication, diabetic peripheral neuropathy (DPN), has a prevalence of 30–50% (3), while osteoporosis (OP) also represents a major public health burden (4, 5). Postmenopausal women with T2DM are at high risk for both conditions, which synergistically increase disability (6–8); however, the underlying comorbidity mechanisms remain unclear. Traditional diagnosis based on bone mineral density (BMD) and symptoms lacks sensitivity (9–11), and classical bone markers fail to explain the “high BMD-high fracture risk” paradox in T2DM or the bone-neuro interplay (12).

Emerging evidence points to Fibroblast Growth Factor 23 (FGF23) and Apelin-13 as key systemic links between metabolism, bone, and nerves. FGF23 regulates phosphorus/vitamin D, inhibits osteoblast differentiation (13, 14), and its axis is implicated in neuroinflammation (15, 16). Apelin-13 improves insulin sensitivity, promotes osteogenesis, inhibits bone resorption, and offers neuroprotection (17–19), suggesting joint involvement in a “bone-endocrine-neuro” axis. However, studies on these factors are often isolated. For postmenopausal T2DM women—characterized by estrogen deficiency, metabolic disorders, abnormal bone turnover, and neural injury risk—systematic evidence is lacking on how FGF23 and Apelin-13 interact with classical bone markers to collectively influence OP and DPN.

Therefore, this cross-sectional study first aims to systematically investigate these correlations in postmenopausal T2DM women. We will: (1) compare indicators across BMD statuses to identify independent BMD factors; (2) analyze independent OP risk/protective factors, evaluating FGF23 and Apelin-13’s predictive value; (3) explore clinical features of OP patients with comorbid T2DM; and (4) analyze independent DPN factors. This study seeks to provide clinical evidence for comorbidity mechanisms and novel early-risk prediction strategies.

## 2 Methods

### 2.1 Study participants

This cross-sectional study enrolled 320 postmenopausal women from our hospital between September 2024 and September 2025. The participants included a T2DM group (*n* = 238) and a non-diabetic OP group (*n* = 82). The study protocol was approved by the Ethics Committee of The First Affiliated Hospital of Kunming Medical University (Approval No.: (2024) Ethics Review L No. 245), and written informed consent was obtained from all participants.

### 2.2 Inclusion and exclusion criteria

1. *Inclusion criteria*: All participants were naturally postmenopausal women (age ≥45) with normal cognitive function and complete data. The T2DM group met the WHO 1999 diagnostic criteria (20). The non-diabetic OP group had no diabetes history and met the Chinese OP diagnostic criteria (21). DPN was diagnosed per the Chinese Diabetes Society 2021 guidelines (22).
2. *Exclusion criteria (All)*: Non-natural menopause/secondary OP; severe systemic disease (cardiac, cerebral, hepatic, renal), active infection, or malignancy; autoimmune diseases; uncontrolled thyroid/parathyroid dysfunction. Additional Exclusion (T2DM group only): Other diabetes types; severe acute diabetic complications; peripheral neuropathy from other causes (e.g., vitamin B12 deficiency).

### 2.3 Study grouping

1. *BMD subgroup analysis*: The T2DM group was stratified by L1-L4 T-score into: Normal bone mass group (T ≥ −1.0, *n* = 83), Osteopenia group (−2.5 < T < −1.0, *n* = 71), and Osteoporosis group (T ≤ −2.5, *n* = 84).
2. *Comorbidity analysis*: ① OP Comorbidity: The T2DM group was divided into Non-OP group (T > −2.5, *n* = 154) and OP group (T ≤ −2.5, *n* = 84). ② T2DM Comorbidity: All postmenopausal OP patients (*n* = 166) were divided into Non-T2DM OP group (*n* = 82) and T2DM with OP group (*n* = 84). ③ DPN Comorbidity: The T2DM group was divided into Non-DPN group (*n* = 58) and DPN group (*n* = 180).

### 2.4 Core measurements and data collection

1. *Clinical data*: Age, diabetes duration (DD), BMI and specific signs for DPN.
2. *Core assessments*: Lumbar spine (L1-L4) BMD (DXA) and nerve function via Current Perception Threshold (CPT) testing (median, superficial peroneal/saphenous nerves).
3. *Laboratory testing*: Standard assays were used for: ① Metabolic Panel: HbA1c, FRUC, glucose/C-peptide/insulin (fasting & postprandial), lipids, liver/renal function, thyroid function, pituitary/sex hormones. ② Bone Metabolism: Serum Ca, Pi, 25OH-VitD, CT, N-MID, *β*-CTX, ALP, PTH. ③ Core Research Indicators: Serum FGF23 and Apelin-13: Serum levels of FGF23 and Apelin-13 were measured using commercially available enzyme-linked immunosorbent assay (ELISA) kits (Shanghai Enzyme-linked Biotechnology Co., Ltd., Shanghai, China). The FGF23 assay employed a kit specific for the intact, biologically active form, while the Apelin-13 assay used a kit specifically recognizing the Apelin-13 isoform. All procedures were performed according to the manufacturer’s instructions, and the absorbance was read using a Rayto RT-6100 microplate reader. The intra-assay coefficient of variation (CV) for FGF23 was <10%, and the inter-assay CV was <15%. For Apelin-13, the intra-assay CV was <10% and the inter-assay CV was <15%. All samples were measured in duplicate, and the mean values were used for subsequent analysis. ④ Glycemic Variability (T2DM only): MAGE, CV, SDBG, TIR (from CGMS). ⑤ Derived Indices: TG/HDL-C, TyG-BMI, HOMA-IR, MHR.

### 2.5 Statistical analysis

Analyses were performed using SPSS 27.0. Continuous data (mean±SD or median[Q_1_, Q_3_]) were compared with *t*-tests, ANOVA, or non-parametric tests. Categorical data (*n*[%]) were compared with Chi-square tests. For comparisons between the non-diabetic OP group (*n* = 82) and the T2DM with OP group (*n* = 84) (Table 1), Mann–Whitney U tests were used, as they are robust to unequal sample sizes and non-normal distributions. Multivariate linear regression identified factors influencing BMD T-scores. Binary logistic regression identified independent risk/protective factors for OP and DPN. Predictive performance was evaluated using ROC curves. *p* < 0.05 was considered significant. This was an exploratory, cross-sectional study. No formal *a priori* sample size calculation was performed. The sample size (*n* = 320) was based on the number of eligible participants who consented during the study period. A post-hoc justification is provided based on the rule of thumb for logistic regression requiring at least 10 events per variable (EPV). For the OP prediction model, there were 84 events (OP cases) and a maximum of 12 candidate variables were considered in the final model, yielding an EPV of 7.0, which is marginally acceptable but carries a risk of overfitting. Therefore, the high AUC values reported for the combined model (e.g., 0.958) should be interpreted cautiously as they may be optimistic due to overfitting. External validation in an independent cohort is essential before any clinical application. For the DPN model, 180 events provided adequate EPV.

**Table 1:** Comparison of clinical indicators between OP patients with and without T2DM.

Variables	Total (*n* = 166)	0 (*n* = 82)	1 (*n* = 84)	Statistic	*p*
Age (year)	64.70 ± 8.94	64.26 ± 8.80	65.13 ± 9.11	*t* = −0.629	0.530
LDL-C (mmol/L)	2.70 ± 0.89	2.61 ± 0.78	2.78 ± 0.99	*t* = −1.196	0.234
TP (g/L)	72.19 ± 6.01	74.86 ± 5.25	69.58 ± 5.58	*t* = 6.277	**<0.001**
BMI (kg/m^2^)	22.46 (21.82, 23.46)	22.46 (22.46, 22.46)	22.70 (21.08, 24.25)	*Z* = −0.779	0.436
HbA1c (%)	6.20 (5.80, 9.78)	5.80 (5.80, 5.80)	9.75 (7.57, 11.33)	*Z* = −11.005	**<0.001**
FRUC (mmol/L)	276.50 (263.20, 375.65)	263.20 (263.20, 263.20)	375.40 (299.75, 433.15)	*Z* = −8.800	**<0.001**
FBG (mmol/L)	5.37 (4.87, 7.16)	5.07 (4.85, 5.45)	7.08 (5.04, 9.06)	*Z* = −5.380	**<0.001**
FCP (ng/mL)	1.69 (1.11, 1.69)	1.69 (1.69, 1.69)	1.21 (0.64, 1.91)	*Z* = −4.251	**<0.001**
FINS (mU/L)	6.90 (6.90, 12.20)	6.90 (6.90, 6.90)	11.43 (7.29, 19.73)	*Z* = −6.862	**<0.001**
2hBG (mmol/L)	8.20 (6.68, 16.36)	6.68 (6.68, 6.68)	16.25 (12.68, 20.57)	*Z* = −11.024	**<0.001**
2hCP (ng/mL)	7.30 (3.24, 7.66)	7.66 (7.66, 7.66)	3.38 (2.15, 5.61)	*Z* = −8.884	**<0.001**
2hINS (mU/L)	50.36 (25.48, 50.36)	50.36 (50.36, 50.36)	26.62 (18.26, 43.78)	*Z* = −6.608	**<0.001**
BUN (mmol/L)	5.54 (4.40, 6.49)	5.54 (4.43, 6.40)	5.23 (4.14, 6.67)	*Z* = −0.413	0.679
Cr (umol/L)	65.55 (58.21, 77.88)	70.10 (62.55, 83.50)	62.40 (55.15, 68.85)	*Z* = −4.065	**<0.001**
UA (umol/L)	292.65 (253.65, 323.75)	299.30 (262.08, 328.05)	289.80 (242.05, 321.38)	*Z* = −1.244	0.214
TC (mmol/L)	4.75 (3.96, 5.23)	4.75 (4.09, 5.17)	4.59 (3.90, 5.34)	*Z* = −0.735	0.462
TG (mmol/L)	1.48 (1.00, 1.85)	1.52 (1.08, 1.67)	1.47 (0.97, 1.97)	*Z* = −0.141	0.888
HDL-C (mmol/L)	1.37 (1.12, 1.57)	1.57 (1.42, 1.70)	1.15 (1.02, 1.35)	*Z* = −7.190	**<0.001**
ALB (g/L)	42.70 (40.15, 44.15)	43.70 (42.32, 45.08)	40.90 (38.55, 43.35)	*Z* = −5.511	**<0.001**
AST (IU/L)	24.00 (19.60, 28.95)	27.60 (23.60, 31.08)	20.70 (17.50, 25.63)	*Z* = −5.095	**<0.001**
ALT (IU/L)	15.55 (10.72, 21.17)	16.50 (12.90, 20.45)	13.65 (9.85, 22.02)	*Z* = −1.822	0.068
WBC (10^9^/L)	6.04 (5.10, 6.67)	6.04 (5.08, 6.21)	5.66 (5.14, 6.85)	*Z* = −0.367	0.714
N (10^9^/L)	3.44 (2.61, 4.00)	3.49 (2.61, 3.49)	3.38 (2.69, 4.16)	*Z* = −0.911	0.363
L (10^9^/L)	2.02 (1.68, 2.27)	2.02 (1.82, 2.17)	2.01 (1.48, 2.38)	*Z* = −0.006	0.995
M (10^9^/L)	0.38 (0.31, 0.44)	0.40 (0.31, 0.41)	0.36 (0.30, 0.46)	*Z* = −0.826	0.409
RBC (10^12^/L)	4.54 (4.30, 4.78)	4.54 (4.47, 4.63)	4.51 (4.18, 4.88)	*Z* = −0.175	0.861
Hb (g/L)	138.00 (131.00, 143.75)	138.00 (136.00, 141.00)	136.00 (128.00, 145.00)	*Z* = −0.717	0.473
T3 (ng/mL)	1.17 (1.00, 1.23)	1.22 (1.09, 1.23)	1.08 (0.90, 1.23)	*Z* = −3.724	**<0.001**
T4 (ng/mL)	78.99 (69.18, 84.67)	78.99 (78.99, 85.97)	74.84 (63.35, 83.50)	*Z* = −2.694	**0.007**
FT3 (pg/mL)	2.88 (2.52, 3.00)	3.00 (2.71, 3.02)	2.66 (2.39, 2.96)	*Z* = −3.787	**<0.001**
FT4 (pg/mL)	12.01 (11.47, 13.02)	12.01 (11.85, 13.14)	12.02 (11.29, 13.00)	*Z* = −0.538	0.591
TSH (uIU/mL)	2.59 (1.95, 3.65)	2.59 (2.01, 3.07)	2.92 (1.85, 4.02)	*Z* = −2.240	**0.025**
Tg (ng/mL)	8.83 (4.00, 18.47)	18.47 (4.04, 18.47)	6.30 (3.91, 16.16)	*Z* = −1.948	0.051
TMAb (IU/mL)	4.79 (0.92, 46.12)	34.53 (1.81, 46.12)	1.65 (0.67, 12.84)	*Z* = −4.032	**<0.001**
TgAb (IU/mL)	25.07 (4.23, 200.67)	197.78 (7.35, 200.67)	8.05 (2.97, 116.10)	*Z* = −3.247	**0.001**
rT3 (ng/mL)	0.52 (0.45, 0.54)	0.53 (0.51, 0.53)	0.48 (0.39, 0.54)	*Z* = −3.833	**<0.001**
TRAb (IU/mL)	0.20 (0.20, 0.20)	0.20 (0.20, 0.20)	0.20 (0.20, 0.20)	*Z* = −2.242	**0.025**
GH (ng/mL)	0.61 (0.13, 0.61)	0.61 (0.61, 0.61)	0.22 (0.09, 0.71)	*Z* = −3.396	**<0.001**
PRL (ng/mL)	16.84 (14.20, 17.75)	16.84 (16.84, 16.84)	15.75 (12.35, 20.63)	*Z* = −0.516	0.606
FSH (mIU/mL)	57.06 (46.50, 57.95)	57.06 (57.06, 57.06)	50.20 (41.17, 64.03)	*Z* = −2.659	**0.008**
LH (mIU/mL)	22.23 (18.02, 22.23)	22.23 (22.23, 22.23)	18.70 (14.60, 23.27)	*Z* = −4.225	**<0.001**
E2 (pg/mL)	15.00 (11.03, 22.00)	22.00 (22.00, 22.00)	11.95 (9.00, 14.32)	*Z* = −8.277	**<0.001**
P (ng/mL)	0.16 (0.10, 0.16)	0.16 (0.16, 0.16)	0.12 (0.10, 0.23)	*Z* = −1.710	0.087
T (ng/mL)	0.07 (0.04, 0.08)	0.08 (0.08, 0.08)	0.04 (0.04, 0.06)	*Z* = −6.124	**<0.001**
Ca (mmol/L)	2.32 (2.27, 2.38)	2.33 (2.28, 2.39)	2.30 (2.24, 2.35)	*Z* = −2.624	**0.009**
Pi (mmol/L)	1.12 (1.02, 1.25)	1.13 (1.01, 1.21)	1.10 (1.03, 1.26)	*Z* = −0.556	0.578
25OH-VitD (nmol/L)	48.22 (39.70, 67.60)	67.28 (53.31, 76.18)	40.71 (34.76, 45.41)	*Z* = −9.141	**<0.001**
CT (pg/mL)	0.40 (0.40, 0.60)	0.40 (0.40, 0.64)	0.40 (0.40, 0.55)	*Z* = −0.925	0.355
N-MID (ng/mL)	13.40 (11.60, 17.28)	16.10 (11.35, 20.17)	12.70 (11.67, 14.95)	*Z* = −2.095	**0.036**
β-CTX (pg/mL)	412.50 (250.25, 577.00)	321.50 (185.25, 520.00)	502.50 (375.25, 644.25)	*Z* = −4.280	**<0.001**
ALP (IU/L)	74.60 (60.90, 89.05)	71.25 (60.15, 87.00)	77.80 (64.03, 92.80)	*Z* = −1.883	0.060
PTH (pg/mL)	47.10 (37.65, 61.73)	46.65 (35.78, 58.85)	48.70 (39.55, 62.42)	*Z* = −0.696	0.486
BMD (g/m^2^)	0.82 (0.78, 0.87)	0.81 (0.74, 0.85)	0.83 (0.80, 0.87)	*Z* = −2.532	**0.011**
T-score	−2.80 (−3.30, −2.60)	−3.00 (−3.58, −2.60)	−2.75 (−3.00, −2.60)	*Z* = −3.223	**0.001**
FGF23 (pg/mL)	423.10 (350.17, 486.02)	370.10 (324.50, 434.72)	462.88 (401.71, 529.45)	*Z* = −5.765	**<0.001**
Apelin-13 (ng/mL)	1.37 (1.11, 1.73)	1.69 (1.43, 1.92)	1.15 (1.03, 1.34)	*Z* = −7.290	**<0.001**
TG/HDL-C	1.06 (0.65, 1.64)	1.05 (0.62, 1.24)	1.29 (0.73, 1.88)	*Z* = −3.011	**0.003**
TyG-BMI	198.02 (184.67, 211.33)	197.93 (185.35, 200.56)	200.09 (184.27, 225.21)	*Z* = −2.016	**0.044**
HOMA-IR	1.73 (1.51, 4.15)	1.55 (1.44, 1.69)	4.08 (2.13, 6.25)	*Z* = −7.307	**<0.001**
MHR	0.27 (0.21, 0.35)	0.25 (0.20, 0.29)	0.31 (0.22, 0.39)	*Z* = −3.825	**<0.001**
Positive or negative for HP (*n*, %)				χ^2^ = 3.593	0.058
0	93 (56.02)	52 (63.41)	41 (48.81)		
1	73 (43.98)	30 (36.59)	43 (51.19)		

*t*, two-sample *t*-test; *Z*, Mann–Whitney U test; χ^2^, Chi-square test; The meaning of the bold values is *P* < 0.05.

## 3 Results

### 3.1 Characteristics of the study population

A total of 320 postmenopausal women were included, comprising the T2DM group (*n* = 238) and the non-diabetic OP group (*n* = 82). In the T2DM group, the median age was 62.0 years, median DD was 10.0 years, median BMI was 23.81 kg/m^2^, and 52.94% had comorbid hypertension (Table 2).

**Table 2:** Comparison of clinical indicators among different bMD subgroups in postmenopausal women with T2DM.

Variables	Total (*n* = 238)	1 (*n* = 83)	2 (*n* = 71)	3 (*n* = 84)	Statistic	*p*
2hBG (mmol/L)	16.99 ± 5.41	16.59 ± 4.94	17.47 ± 5.50	16.97 ± 5.79	*F* = 0.500	0.607
TC (mmol/L)	4.62 ± 1.13	4.64 ± 1.15	4.57 ± 1.21	4.63 ± 1.06	*F* = 0.092	0.912
LDL-C (mmol/L)	2.68 ± 0.97	2.60 ± 0.91	2.66 ± 1.03	2.78 ± 0.99	*F* = 0.699	0.498
TP (g/L)	70.46 ± 5.85	71.82 ± 5.78	69.92 ± 6.04	69.58 ± 5.58	*F* = 3.585	**0.029**
RBC (10^12^/L)	4.54 ± 0.51	4.59 ± 0.50	4.52 ± 0.53	4.52 ± 0.52	*F* = 0.439	0.645
T4 (ng/mL)	74.48 ± 15.67	74.72 ± 16.44	74.00 ± 15.84	74.64 ± 14.90	*F* = 0.047	0.954
Ca (mmol/L)	2.32 ± 0.10	2.34 ± 0.10	2.31 ± 0.11	2.30 ± 0.10	*F* = 3.269	**0.040**
Pi (mmol/L)	1.17 ± 0.18	1.20 ± 0.18	1.17 ± 0.19	1.15 ± 0.18	*F* = 1.571	0.210
Age (year)	62.00 (56.00, 70.00)	60.00 (54.00, 66.00)	61.00 (57.50, 69.50)	65.00 (58.00, 72.00)	H = 12.232	**0.002**
DD (year)	10.00 (4.00, 16.00)	5.00 (1.00, 10.00)	10.00 (3.50, 15.00)	15.00 (10.00, 20.00)	H = 65.710	**<0.001**
BMI (kg/m^2^)	23.81 (21.91, 26.21)	25.44 (23.34, 27.88)	23.62 (21.94, 25.84)	22.70 (21.08, 24.25)	H = 35.499	**<0.001**
HbA1c (%)	8.20 (6.80, 10.23)	7.50 (6.65, 8.55)	7.80 (6.75, 10.25)	9.75 (7.57, 11.33)	H = 29.273	**<0.001**
FRUC (mmol/L)	328.20 (277.33, 390.60)	309.30 (272.55, 348.50)	314.30 (273.80, 389.65)	375.40 (299.75, 433.15)	H = 19.571	**<0.001**
FBG (mmol/L)	6.27 (5.02, 8.34)	6.05 (5.03, 7.38)	6.13 (5.00, 8.02)	7.08 (5.04, 9.06)	H = 4.442	0.109
FCP (ng/mL)	1.38 (0.81, 2.02)	1.50 (0.93, 2.08)	1.35 (0.85, 2.08)	1.21 (0.64, 1.91)	H = 2.800	0.247
FINS (mU/L)	13.08 (8.94, 20.21)	14.29 (9.15, 21.41)	13.14 (9.53, 20.78)	11.43 (7.29, 19.73)	H = 3.805	0.149
2hCP (ng/mL)	3.61 (2.30, 5.58)	3.94 (2.40, 5.65)	3.61 (2.33, 5.25)	3.38 (2.15, 5.61)	H = 0.784	0.676
2hINS (mU/L)	29.86 (20.31, 44.87)	32.68 (22.21, 48.36)	34.06 (20.21, 43.87)	26.62 (18.26, 43.78)	H = 3.995	0.136
BUN (mmol/L)	5.36 (4.20, 6.96)	5.54 (4.17, 6.66)	5.32 (4.30, 7.07)	5.23 (4.14, 6.67)	H = 0.707	0.702
Cr (umol/L)	62.55 (54.93, 71.70)	62.50 (55.80, 73.60)	63.70 (53.35, 72.85)	62.40 (55.15, 68.85)	H = 0.590	0.745
UA (umol/L)	297.70 (252.08, 354.85)	305.50 (261.65, 371.40)	311.70 (265.45, 358.85)	289.80 (242.05, 321.38)	H = 6.112	**0.047**
TG (mmol/L)	1.48 (1.06, 2.26)	1.61 (1.06, 2.28)	1.37 (1.16, 2.42)	1.47 (0.97, 1.97)	H = 3.290	0.193
HDL-C (mmol/L)	1.12 (1.00, 1.33)	1.12 (0.98, 1.33)	1.09 (0.97, 1.30)	1.15 (1.02, 1.35)	H = 2.279	0.320
ALB (g/L)	41.30 (39.58, 43.50)	42.50 (40.45, 43.65)	41.10 (39.50, 43.05)	40.90 (38.55, 43.35)	H = 7.321	**0.026**
AST (IU/L)	21.15 (17.60, 26.20)	22.40 (17.80, 26.80)	20.50 (17.25, 25.14)	20.70 (17.50, 25.63)	H = 1.553	0.460
ALT (IU/L)	16.30 (10.38, 24.08)	17.50 (13.35, 25.15)	17.20 (9.79, 24.50)	13.65 (9.85, 22.02)	H = 6.865	**0.032**
WBC (10^9^/L)	5.69 (4.91, 6.78)	5.72 (4.81, 6.93)	5.59 (4.87, 6.58)	5.66 (5.14, 6.85)	H = 1.210	0.546
N (10^9^/L)	3.26 (2.44, 4.02)	3.09 (2.30, 4.07)	3.15 (2.41, 3.71)	3.38 (2.69, 4.16)	H = 3.790	0.150
L (10^9^/L)	2.03 (1.64, 2.40)	2.10 (1.71, 2.45)	1.94 (1.61, 2.33)	2.01 (1.48, 2.38)	H = 3.132	0.209
M (10^9^/L)	0.37 (0.30, 0.46)	0.38 (0.30, 0.47)	0.34 (0.28, 0.50)	0.36 (0.30, 0.46)	H = 0.557	0.757
Hb (g/L)	137.50 (128.00, 146.00)	139.00 (129.00, 147.50)	136.00 (128.00, 144.50)	136.00 (128.00, 145.00)	H = 1.223	0.543
T3 (ng/mL)	1.03 (0.89, 1.19)	1.03 (0.90, 1.17)	1.01 (0.88, 1.15)	1.08 (0.90, 1.23)	H = 2.524	0.283
FT3 (pg/mL)	2.66 (2.35, 2.93)	2.70 (2.41, 2.95)	2.58 (2.31, 2.88)	2.66 (2.39, 2.96)	H = 2.135	0.344
FT4 (pg/mL)	12.11 (11.14, 13.35)	12.10 (11.11, 13.40)	12.44 (11.08, 13.47)	12.02 (11.29, 13.00)	H = 0.605	0.739
TSH (uIU/mL)	2.95 (1.84, 4.25)	2.98 (1.88, 4.46)	2.95 (1.68, 4.28)	2.92 (1.85, 4.02)	H = 0.248	0.883
Tg (ng/mL)	6.52 (3.58, 14.25)	5.84 (3.48, 11.76)	7.54 (4.25, 15.32)	6.30 (3.91, 16.16)	H = 1.582	0.453
TMAb (IU/mL)	1.69 (0.67, 9.49)	2.09 (0.84, 6.06)	1.42 (0.57, 8.20)	1.65 (0.67, 12.84)	H = 0.564	0.754
TgAb (IU/mL)	8.04 (3.07, 92.33)	9.33 (3.54, 50.50)	7.20 (2.90, 91.31)	8.05 (2.97, 116.10)	H = 0.126	0.939
rT3 (ng/mL)	0.48 (0.40, 0.54)	0.49 (0.41, 0.55)	0.46 (0.40, 0.54)	0.48 (0.39, 0.54)	H = 1.954	0.376
TRAb (IU/L)	0.20 (0.20, 0.20)	0.20 (0.20, 0.26)	0.20 (0.20, 0.20)	0.20 (0.20, 0.20)	H = 3.787	0.151
GH (ng/mL)	0.17 (0.08, 0.56)	0.16 (0.07, 0.43)	0.18 (0.11, 0.50)	0.22 (0.09, 0.71)	H = 2.383	0.304
PRL (ng/mL)	16.21 (12.48, 20.71)	16.35 (13.02, 20.39)	16.18 (11.71, 21.02)	15.75 (12.35, 20.63)	H = 0.844	0.656
FSH (mIU/mL)	51.40 (37.35, 64.03)	51.10 (33.35, 62.60)	55.70 (36.85, 66.20)	50.20 (41.17, 64.03)	H = 1.292	0.524
LH (mIU/mL)	18.65 (13.58, 24.83)	17.17 (12.25, 25.70)	19.10 (14.65, 26.50)	18.70 (14.60, 23.27)	H = 1.757	0.415
E2 (pg/mL)	12.65 (10.40, 15.45)	13.20 (10.80, 16.35)	12.90 (10.85, 15.75)	11.95 (9.00, 14.32)	H = 6.658	**0.036**
P (ng/mL)	0.13 (0.10, 0.22)	0.10 (0.10, 0.21)	0.15 (0.10, 0.21)	0.12 (0.10, 0.23)	H = 1.812	0.404
T (ng/mL)	0.04 (0.04, 0.07)	0.05 (0.04, 0.08)	0.04 (0.04, 0.08)	0.04 (0.04, 0.06)	H = 4.712	0.095
25OH-VitD (nmol/L)	44.51 (38.19, 55.40)	55.49 (44.62, 64.38)	43.88 (36.24, 53.55)	40.71 (34.76, 45.41)	H = 52.974	**<0.001**
CT (pg/mL)	0.40 (0.40, 0.58)	0.40 (0.40, 0.69)	0.40 (0.40, 0.40)	0.40 (0.40, 0.55)	H = 4.084	0.130
N-MID (ng/mL)	13.60 (12.10, 16.33)	15.40 (13.20, 17.35)	13.40 (12.60, 14.60)	12.70 (11.67, 14.95)	H = 17.011	**<0.001**
β-CTX (pg/mL)	421.00 (309.00, 541.00)	360.00 (268.00, 476.50)	421.00 (338.50, 523.50)	502.50 (375.25, 644.25)	H = 18.050	**<0.001**
ALP (IU/L)	75.85 (62.45, 93.70)	73.40 (60.25, 90.35)	75.90 (65.90, 98.30)	77.80 (64.03, 92.80)	H = 1.289	0.525
PTH (pg/mL)	41.75 (32.98, 52.65)	37.00 (30.00, 43.15)	40.80 (32.55, 50.40)	48.70 (39.55, 62.42)	H = 24.601	**<0.001**
MAGE (mmol/L)	4.85 (3.61, 6.61)	4.73 (3.58, 5.92)	4.93 (3.68, 6.67)	5.10 (3.90, 7.00)	H = 1.952	0.377
CV (%)	25.04 (20.48, 31.38)	22.51 (20.05, 27.99)	25.60 (21.36, 31.84)	25.69 (20.61, 34.12)	H = 9.273	**0.010**
MBG (mmol/L)	9.11 (7.56, 10.66)	8.79 (7.53, 10.43)	9.30 (8.20, 10.88)	9.26 (7.40, 10.62)	H = 2.965	0.227
SDBG (mmol/L)	2.45 (1.96, 3.20)	2.30 (1.88, 2.81)	2.54 (2.08, 3.31)	2.58 (2.00, 3.36)	H = 4.507	0.105
TIR (%)	68.76 (48.81, 84.23)	73.43 (55.11, 85.73)	65.66 (35.00, 78.46)	67.88 (51.90, 82.47)	H = 4.182	0.124
CPT (median nerve) (score)	8.37 (5.00, 9.41)	5.00 (2.50, 8.13)	8.41 (7.00, 9.37)	9.37 (8.37, 9.78)	H = 63.436	**<0.001**
CPT (sural/saphenous nerve) (score)	7.00 (0.00, 8.75)	0.00 (0.00, 3.00)	3.00 (0.00, 8.00)	8.76 (8.00, 9.37)	H = 91.836	**<0.001**
BMD (g/m^2^)	0.99 (0.87, 1.12)	1.16 (1.11, 1.25)	0.99 (0.94, 1.03)	0.83 (0.80, 0.87)	H = 206.782	**<0.001**
T-score	−1.50 (−2.60, −0.50)	−0.20 (−0.60, 0.60)	−1.50 (−1.80, −1.20)	−2.75 (−3.00, −2.60)	H = 210.359	**<0.001**
FGF23 (pg/mL)	426.63 (361.48, 486.17)	381.26 (307.42, 439.59)	425.69 (362.61, 485.04)	462.88 (401.71, 529.45)	H = 33.566	**<0.001**
Apelin-13 (ng/mL)	1.53 (1.17, 2.04)	2.11 (1.87, 2.44)	1.48 (1.31, 1.85)	1.15 (1.03, 1.34)	H = 129.013	**<0.001**
TG/HDL-C	1.34 (0.87, 2.22)	1.42 (0.88, 2.31)	1.35 (0.92, 2.69)	1.29 (0.73, 1.88)	H = 2.860	0.239
TyG-BMI	213.48 (192.20, 238.02)	221.98 (203.45, 255.32)	214.50 (192.65, 236.80)	200.09 (184.27, 225.21)	H = 25.202	**<0.001**
HOMA-IR	3.92 (2.36, 6.52)	3.81 (2.58, 6.02)	4.03 (2.31, 7.27)	4.08 (2.13, 6.25)	H = 0.666	0.717
MHR	0.33 (0.25, 0.42)	0.34 (0.27, 0.46)	0.33 (0.24, 0.42)	0.31 (0.22, 0.39)	H = 1.970	0.373
Positive or Negative for HP (*n*, %)					χ^2^ = 2.510	0.285
0	112 (47.06)	43 (51.81)	28 (39.44)	41 (48.81)		
1	126 (52.94)	40 (48.19)	43 (60.56)	43 (51.19)		

F, ANOVA; H, Kruskal–Wallis test; χ^2^, Chi-square test; The meaning of the bold values is *P* < 0.05.

### 3.2 Relationship between BMD status and serum markers/nerve function in postmenopausal women with T2DM

T2DM patients were stratified by BMD into three groups: Normal bone mass (*n* = 83), Osteopenia (*n* = 71), and Osteoporosis (*n* = 84). As shown in Table 2, as the T-score progressively decreased, serum levels of BMI, 25OH-VitD, N-MID, Apelin-13, and TyG-BMI showed a stepwise decline (*p* < 0.001); Conversely, DD, HbA1c, FRUC, *β*-CTX, PTH, FGF23, and CPT scores increased sequentially (*p* < 0.001).

**Figure 1:**
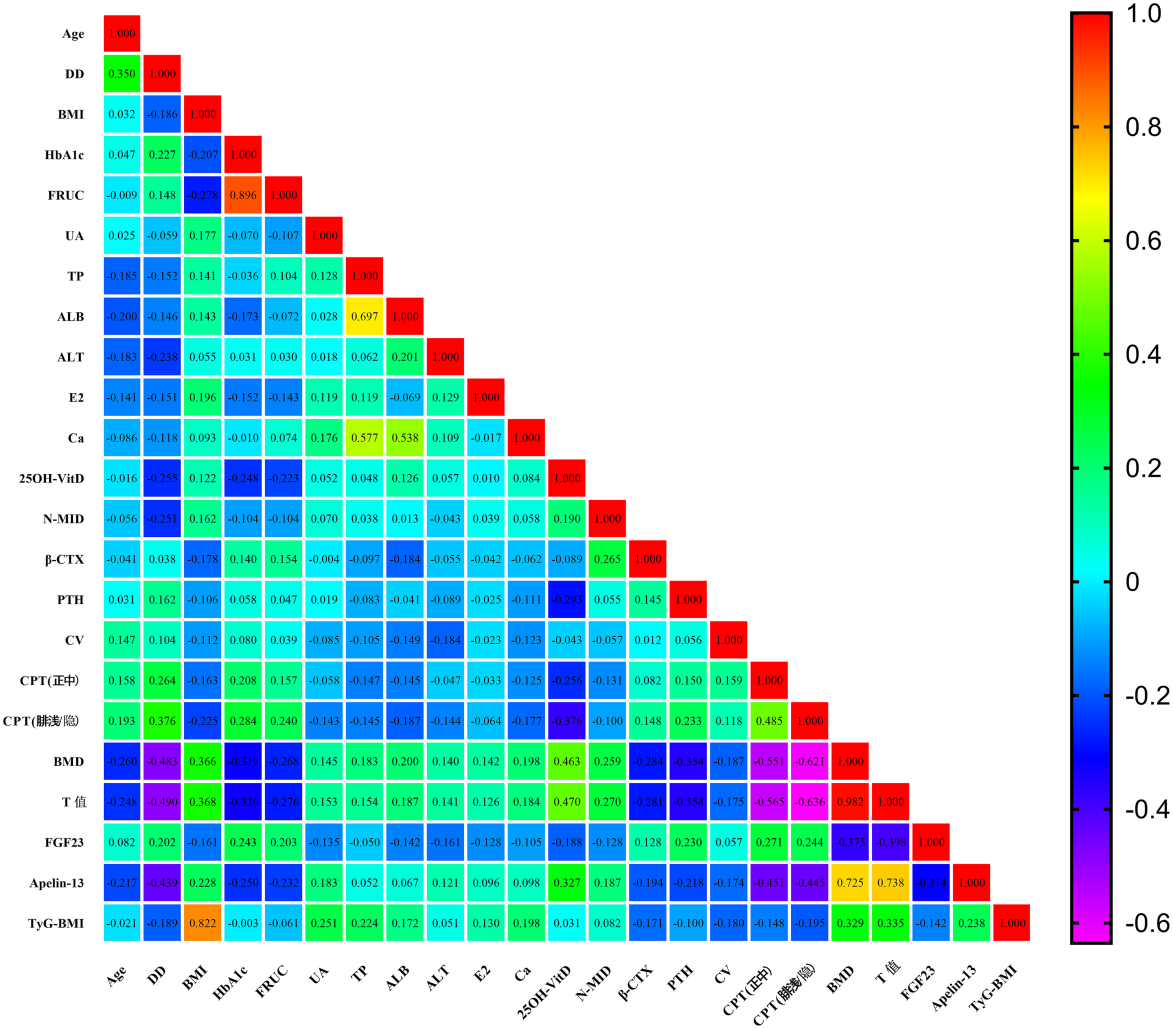
Spearman correlation heatmap of significant clinical indicators among three groups.

Given that the T-score was not normally distributed, Spearman correlation analysis was performed to assess its univariate correlations with other clinical indicators that showed statistically significant differences among the three groups. As shown in Table 3 and Figure 1, the T-score was significantly negatively correlated with Age, DD, HbA1c, FRUC, *β*-CTX, PTH, CV, CPT (median nerve), CPT (superficial peroneal/saphenous nerve), and FGF23 (all *p* < 0.05). It was significantly positively correlated with BMI, UA, TP, ALB, ALT, Ca, 25OH-VitD, N-MID, BMD, Apelin-13, and TyG-BMI (all *p* < 0.05). No significant correlation was observed with E2.

**Table 3:** Spearman correlation analysis of T-score with other clinical indicators.

Variables	T-score	
	ρ	*p*
Age	−0.248**	**<0.001**
DD	−0.490**	**<0.001**
BMI	0.368**	**<0.001**
HbA1c	−0.326**	**<0.001**
FRUC	−0.276**	**<0.001**
UA	0.153*	**0.018**
TP	0.154*	**0.017**
ALB	0.187**	**0.004**
ALT	0.141*	**0.030**
E2	0.126	0.052
Ca	0.184**	**0.004**
25OH-VitD	0.470**	**<0.001**
N-MID	0.270**	**<0.001**
β-CTX	−0.281**	**<0.001**
PTH	−0.354**	**<0.001**
CV	−0.175**	**0.007**
CPT (median nerve)	−0.565**	**<0.001**
CPT (sural/saphenous nerve)	−0.636**	**<0.001**
BMD	0.982**	**<0.001**
T-score	1.000	**-**
FGF23	−0.398**	**<0.001**
Apelin-13	0.738**	**<0.001**
TyG-BMI	0.335**	**<0.001**

*p < 0.05, **p < 0.01, ***p < 0.001; The meaning of the bold values is *P* < 0.05.

**Figure 2:**
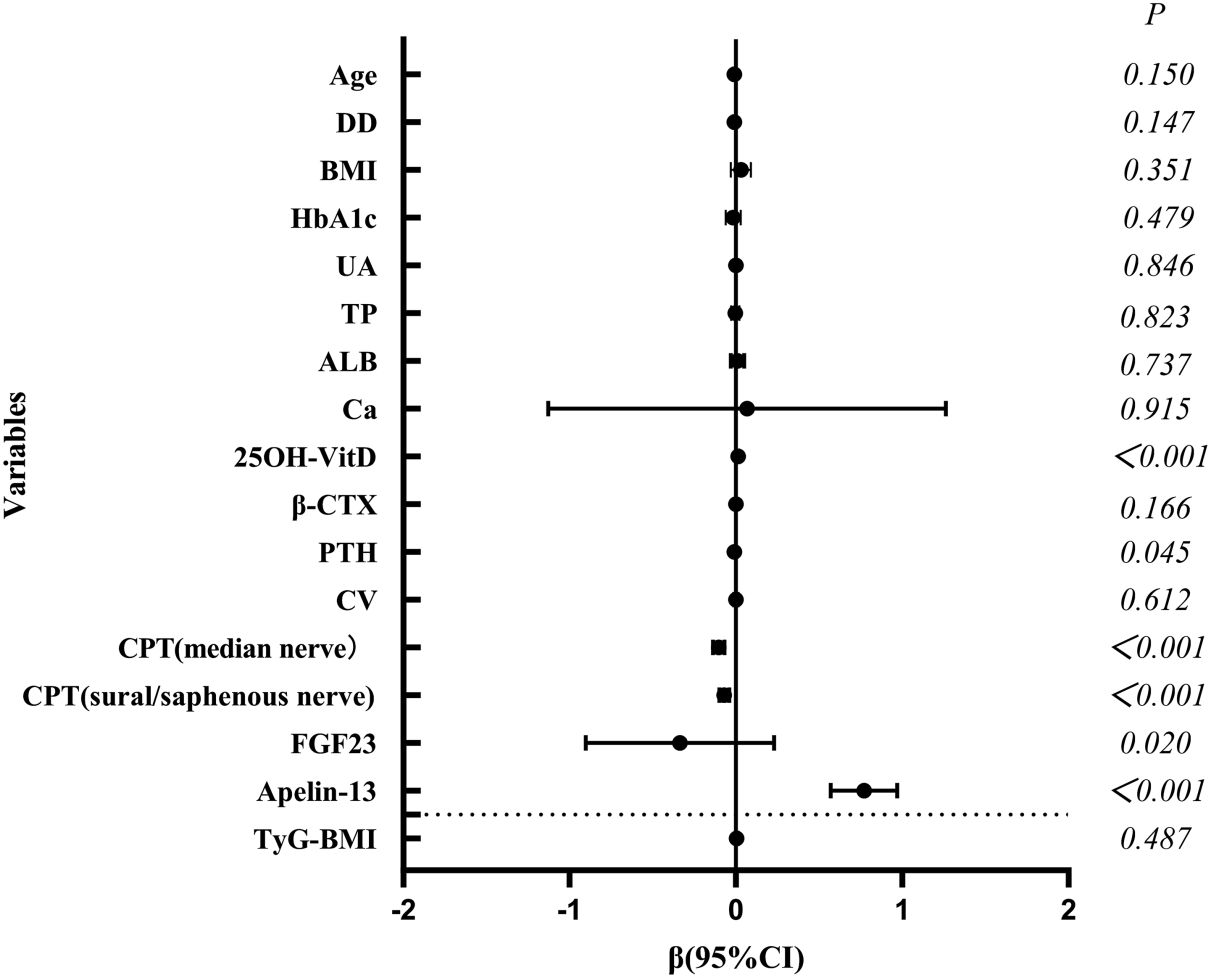
Forest plot of independent factors influencing the T-score (multiple linear regression).

As shown in Table 4 and Figure 2, multivariate linear regression confirmed Apelin-13 and 25OH-VitD as independent protective factors for the T-score, while FGF23, PTH, and CPT scores were independent risk factors (all *p* < 0.05).

**Table 4:** Linear regression analysis of factors influencing the T-score.

Variables	Univariate					Multivariate				
	*β*	S. E	*t*	*p*	*β* (95%CI)	*β*	S. E	*t*	*p*	β (95%CI)
Age	s	0.01	−3.99	**<0.001**	−0.04 (−0.06 ~ −0.02)	−0.01	0.01	−1.44	0.150	−0.01 (−0.02 ~ 0.00)
DD	−0.08	0.01	−7.70	**<0.001**	−0.08 (−0.10 ~ −0.06)	−0.01	0.01	−1.45	0.147	−0.01 (−0.02 ~ 0.00)
BMI	0.15	0.02	5.92	**<0.001**	0.15 (0.10 ~ 0.20)	0.03	0.03	0.93	0.351	0.03 (−0.03 ~ 0.09)
HbA1c	−0.20	0.04	−5.48	**<0.001**	−0.20 (−0.27 ~ −0.13)	−0.02	0.02	−0.71	0.479	−0.02 (−0.06 ~ 0.03)
UA	0.01	0.00	2.57	**0.011**	0.01 (0.01 ~ 0.01)	0.00	0.00	0.19	0.846	0.00 (−0.00 ~ 0.00)
TP	0.04	0.02	2.53	**0.012**	0.04 (0.01 ~ 0.07)	−0.00	0.01	−0.22	0.823	−0.00 (−0.03 ~ 0.02)
ALB	0.07	0.02	2.99	**0.003**	0.07 (0.02 ~ 0.12)	0.01	0.02	0.34	0.737	0.01 (−0.03 ~ 0.05)
ALT	0.01	0.01	1.57	0.118	0.01 (−0.00 ~ 0.02)					
Ca	2.21	0.85	2.60	**0.010**	2.21 (0.54 ~ 3.88)	0.07	0.61	0.11	0.915	0.07 (−1.13 ~ 1.26)
25OH-VitD	0.04	0.01	7.76	**<0.001**	0.04 (0.03 ~ 0.05)	0.01	0.00	3.76	**<0.001**	0.01 (0.01 ~ 0.02)
N-MID	0.01	0.01	1.11	0.268	0.01 (−0.01 ~ 0.04)					
β-CTX	−0.01	0.00	−3.65	**<0.001**	−0.01 (−0.99 ~ −0.01)	−0.00	0.00	−1.39	0.166	−0.00 (−0.00 ~ 0.00)
PTH	−0.02	0.00	−4.95	**<0.001**	−0.02 (−0.03 ~ −0.01)	−0.01	0.00	−2.02	**0.045**	−0.01 (−0.01 ~ −0.01)
CV	−0.03	0.01	−2.74	**0.007**	−0.03 (−0.05 ~ −0.01)	−0.00	0.01	−0.51	0.612	−0.00 (−0.01 ~ 0.01)
CPT (median nerve)	−0.27	0.02	−12.02	**<0.001**	−0.27 (−0.31 ~ −0.22)	−0.10	0.02	−5.71	**<0.001**	−0.10 (−0.14 ~ −0.07)
CPT (sural/saphenous nerve)	−0.22	0.02	−13.00	**<0.001**	−0.22 (−0.25 ~ −0.19)	−0.07	0.02	−4.41	**<0.001**	−0.07 (−0.10 ~ −0.04)
FGF23	−0.01	0.00	−6.56	**<0.001**	−0.01 (−0.99 ~ −0.01)	−0.01	0.00	−2.34	**0.020**	−0.01 (−0.99 ~ −0.01)
Apelin-13	1.60	0.11	14.34	**<0.001**	1.60 (1.38 ~ 1.82)	0.77	0.10	7.54	**<0.001**	0.77 (0.57 ~ 0.97)
TyG-BMI	0.01	0.00	5.71	**<0.001**	0.01 (0.01 ~ 0.02)	0.00	0.00	0.70	0.487	0.00 (−0.00 ~ 0.01)

The model ANOVA was significant (*p* < 0.001). The VIF for all independent variables was less than 5. The adjusted R^2^ was 0.720, indicating a good model fit; The meaning of the bold values is *P* < 0.05.

### 3.3 Risk factors and predictive model for OP in postmenopausal women with T2DM

As shown in Table 5, the OP group had significantly higher levels of Age, DD, HbA1c, FRUC, *β*-CTX, PTH, CPT scores and FGF23, and lower levels of BMI, 25OH-VitD, N-MID, Apelin-13 and TyG-BMI (all *p* < 0.05) compared to the Non-OP group.

**Table 5:** Comparison of clinical indicators between T2DM patients with and without OP.

Variables	Total (*n* = 238)	0 (*n* = 154)	1 (*n* = 84)	Statistic	*p*
2hBG (mmol/L)	16.99 ± 5.41	16.99 ± 5.21	16.97 ± 5.79	*t* = 0.029	0.977
TC (mmol/L)	4.62 ± 1.13	4.61 ± 1.17	4.63 ± 1.06	*t* = −0.123	0.902
LDL-C (mmol/L)	2.68 ± 0.97	2.63 ± 0.96	2.78 ± 0.99	*t* = −1.118	0.265
TP (g/L)	70.46 ± 5.85	70.94 ± 5.96	69.58 ± 5.58	*t* = 1.729	0.085
RBC (10^12^/L)	4.54 ± 0.51	4.56 ± 0.51	4.52 ± 0.52	*t* = 0.525	0.600
T4 (ng/mL)	74.48 ± 15.67	74.39 ± 16.12	74.64 ± 14.90	*t* = −0.121	0.904
Pi (mmol/L)	1.17 ± 0.18	1.18 ± 0.19	1.15 ± 0.18	*t* = 1.564	0.119
Age (year)	62.00 (56.00, 69.75)	61.00 (55.00, 67.00)	65.00 (58.00, 72.00)	*Z* = −2.994	**0.003**
DD (year)	10.00 (4.00, 15.75)	7.00 (2.00, 10.75)	15.00 (10.00, 20.00)	*Z* = −7.219	**<0.001**
BMI (kg/m^2^)	23.81 (21.93, 26.17)	24.73 (22.60, 27.20)	22.70 (21.08, 24.25)	*Z* = −5.067	**<0.001**
HbA1c (%)	8.20 (6.80, 10.20)	7.60 (6.70, 9.38)	9.75 (7.57, 11.33)	*Z* = −4.772	**<0.001**
FRUC (mmol/L)	328.20 (277.57, 390.27)	311.35 (272.43, 362.40)	375.40 (299.75, 433.15)	*Z* = −4.164	**<0.001**
FBG (mmol/L)	6.26 (5.04, 8.30)	6.06 (4.99, 7.62)	7.08 (5.04, 9.06)	*Z* = −1.913	0.056
FCP (ng/mL)	1.38 (0.81, 2.02)	1.48 (0.86, 2.09)	1.21 (0.64, 1.91)	*Z* = −1.614	0.107
FINS (mU/L)	13.08 (9.02, 20.14)	13.85 (9.32, 21.34)	11.43 (7.29, 19.73)	*Z* = −1.931	0.054
2hCP (ng/mL)	3.60 (2.30, 5.57)	3.81 (2.35, 5.52)	3.38 (2.15, 5.61)	*Z* = −0.590	0.555
2hINS (mU/L)	29.86 (20.32, 44.61)	33.33 (22.07, 45.66)	26.62 (18.26, 43.78)	*Z* = −1.878	0.060
BUN (mmol/L)	5.36 (4.20, 6.94)	5.49 (4.24, 6.98)	5.23 (4.14, 6.67)	*Z* = −0.499	0.617
Cr (umol/L)	62.55 (55.00, 71.70)	62.95 (54.78, 73.70)	62.40 (55.15, 68.85)	*Z* = −0.749	0.454
UA (umol/L)	297.70 (253.05, 354.73)	309.85 (262.85, 364.30)	289.80 (242.05, 321.38)	*Z* = −2.455	**0.014**
TG (mmol/L)	1.48 (1.06, 2.26)	1.50 (1.09, 2.33)	1.47 (0.97, 1.97)	*Z* = −1.796	0.073
HDL-C (mmol/L)	1.12 (1.00, 1.33)	1.10 (0.98, 1.32)	1.15 (1.02, 1.35)	*Z* = −1.403	0.161
ALB (g/L)	41.30 (39.60, 3.50)	41.50 (39.97, 43.58)	40.90 (38.55, 43.35)	*Z* = −1.887	0.059
AST (IU/L)	21.15 (17.60, 6.03)	21.25 (17.60, 26.50)	20.70 (17.50, 25.63)	*Z* = −0.520	0.603
ALT (IU/L)	16.30 (10.43, 4.00)	17.30 (11.88, 24.85)	13.65 (9.85, 22.02)	*Z* = −2.296	**0.022**
WBC (10^9^/L)	5.69 (4.91, 6.77)	5.71 (4.83, 6.70)	5.66 (5.14, 6.85)	*Z* = −0.998	0.318
N (10^9^/L)	3.26 (2.44, 4.01)	3.11 (2.38, 3.88)	3.38 (2.69, 4.16)	*Z* = −1.933	0.053
L (10^9^/L)	2.03 (1.64, 2.40)	2.03 (1.66, 2.41)	2.01 (1.48, 2.38)	*Z* = −0.379	0.704
M (10^9^/L)	0.37 (0.30, 0.46)	0.37 (0.30, 0.49)	0.36 (0.30, 0.46)	*Z* = −0.270	0.787
Hb (g/L)	137.50 (128.00, 145.75)	138.00 (128.25, 146.00)	136.00 (128.00, 145.00)	*Z* = −0.602	0.547
T3 (ng/mL)	1.03 (0.89, 1.19)	1.01 (0.89, 1.16)	1.08 (0.90, 1.23)	*Z* = −1.477	0.140
FT3 (pg/mL)	2.66 (2.35, 2.93)	2.66 (2.35, 2.92)	2.66 (2.39, 2.96)	*Z* = −0.634	0.526
FT4 (pg/mL)	12.11 (11.15, 13.33)	12.23 (11.10, 13.43)	12.02 (11.29, 13.00)	*Z* = −0.561	0.575
TSH (uIU/mL)	2.95 (1.85, 4.23)	2.96 (1.85, 4.37)	2.92 (1.85, 4.02)	*Z* = −0.366	0.714
Tg (ng/mL)	6.52 (3.59, 14.24)	6.64 (3.57, 13.45)	6.30 (3.91, 16.16)	*Z* = −0.360	0.719
TMAb (IU/mL)	1.69 (0.67, 9.33)	1.73 (0.72, 6.56)	1.65 (0.67, 12.84)	*Z* = −0.455	0.649
TgAb (IU/mL)	8.04 (3.07, 91.76)	8.00 (3.23, 75.29)	8.05 (2.97, 116.10)	*Z* = −0.352	0.725
rT3 (ng/mL)	0.48 (0.40, 0.54)	0.48 (0.40, 0.54)	0.48 (0.39, 0.54)	*Z* = −0.119	0.905
TRAb (IU/L)	0.20 (0.20, 0.20)	0.20 (0.20, 0.20)	0.20 (0.20, 0.20)	*Z* = −0.385	0.701
GH (ng/mL)	0.17 (0.08, 0.56)	0.17 (0.08, 0.51)	0.22 (0.09, 0.71)	*Z* = −1.170	0.242
PRL (ng/mL)	16.21 (12.52, 20.68)	16.34 (12.60, 20.70)	15.75 (12.35, 20.63)	*Z* = −0.611	0.541
FSH (mIU/mL)	51.40 (37.35, 63.88)	52.61 (35.32, 63.48)	50.20 (41.17, 64.03)	*Z* = −0.400	0.689
LH (mIU/mL)	18.65 (13.60, 24.75)	18.55 (12.90, 26.20)	18.70 (14.60, 23.27)	*Z* = −0.013	0.990
E2 (pg/mL)	12.65 (10.40, 15.40)	12.95 (10.83, 15.97)	11.95 (9.00, 14.32)	*Z* = −2.495	**0.013**
P (ng/mL)	0.13 (0.10, 0.22)	0.13 (0.10, 0.21)	0.12 (0.10, 0.23)	*Z* = −0297	0.766
T (ng/mL)	0.04 (0.04, 0.07)	0.04 (0.04, 0.08)	0.04 (0.04, 0.06)	*Z* = −1.956	0.051
Ca (mmol/L)	2.32 (2.25, 2.39)	2.33 (2.26, 2.39)	2.30 (2.24, 2.35)	*Z* = −2.086	**0.037**
25OH-VitD (nmol/L)	44.51 (38.39, 55.28)	50.33 (41.25, 60.50)	40.71 (34.76, 45.41)	*Z* = −5.824	**<0.001**
CT (pg/mL)	0.40 (0.40, 0.58)	0.40 (0.40, 0.59)	0.40 (0.40, 0.55)	*Z* = −0.476	0.634
N-MID (ng/mL)	13.60 (12.10, 16.28)	14.20 (12.80, 16.65)	12.70 (11.67, 14.95)	*Z* = −3.048	**0.002**
β-CTX (pg/mL)	421.00 (310.25, 540.75)	398.00 (296.00, 505.75)	502.50 (375.25, 644.25)	*Z* = −3.701	**<0.001**
ALP (IU/L)	75.85 (63.12, 93.62)	75.30 (61.35, 94.00)	77.80 (64.03, 92.80)	*Z* = −0.494	0.622
PTH (pg/mL)	41.75 (33.00, 52.45)	37.75 (31.10, 47.75)	48.70 (39.55, 62.42)	*Z* = −4.637	**<0.001**
MAGE (mmol/L)	4.85 (3.62, 6.61)	4.77 (3.60, 6.55)	5.10 (3.90, 7.00)	*Z* = −0.721	0.471
CV (%)	25.04 (20.50, 31.35)	24.19 (20.50, 29.40)	25.69 (20.61, 34.12)	*Z* = −1.824	0.068
MBG (mmol/L)	9.11 (7.58, 10.65)	9.09 (7.90, 10.65)	9.26 (7.40, 10.62)	*Z* = −0.060	0.952
SDBG (mmol/L)	2.45 (1.96, 3.19)	2.32 (1.96, 3.12)	2.58 (2.00, 3.36)	*Z* = −0.944	0.345
TIR (%)	68.76 (49.40, 83.86)	69.29 (48.33, 84.62)	67.88 (51.90, 82.47)	*Z* = −0.125	0.900
CPT (median nerve) (score)	8.37 (5.00, 9.40)	7.37 (3.00, 9.00)	9.37 (8.37, 9.78)	*Z* = −6.640	**<0.001**
CPT (sural/saphenous nerve) (score)	7.00 (0.00, 8.74)	0.00 (0.00, 7.28)	8.76 (8.00, 9.37)	*Z* = −9.329	**<0.001**
BMD (g/m^2^)	0.99 (0.87, 1.12)	1.08 (1.00, 1.16)	0.83 (0.80, 0.87)	*Z* = −12.546	**<0.001**
T-score	−1.50 (−2.60, −0.50)	−0.85 (−1.50, −0.20)	−2.75 (−3.00, −2.60)	*Z* = −12.751	**<0.001**
FGF23 (pg/mL)	426.63 (361.48, 486.17)	411.59 (328.77, 461.96)	462.88 (401.71, 529.45)	*Z* = −5.131	**<0.001**
Apelin-13 (ng/mL)	1.53 (1.17, 2.04)	1.88 (1.48, 2.25)	1.15 (1.03, 1.34)	*Z* = −9.575	**<0.001**
TG/HDL-C	1.34 (0.87, 2.20)	1.39 (0.89, 2.50)	1.29 (0.73, 1.88)	*Z* = −1.682	0.092
TyG-BMI	213.48 (192.22, 237.77)	219.41 (198.61, 248.00)	200.09 (184.27, 225.21)	*Z* = −4.409	**<0.001**
HOMA-IR	3.92 (2.38, 6.49)	3.86 (2.48, 6.78)	4.08 (2.13, 6.25)	*Z* = −0.731	0.465
MHR	0.33 (0.25, 0.42)	0.34 (0.26, 0.43)	0.31 (0.22, 0.39)	*Z* = −1.288	0.198
Positive or Negative for HP (*n*, %)				χ^2^ = 0.160	0.689
0	112 (47.06)	71 (46.10)	41 (48.81)		
1	126 (52.94)	83 (53.90)	43 (51.19)		

*t*, two-sample *t*-test; *Z*, Mann–Whitney U test; *χ*^2^, Chi-square test; The meaning of the bold values is *P* < 0.05.

**Figure 3:**
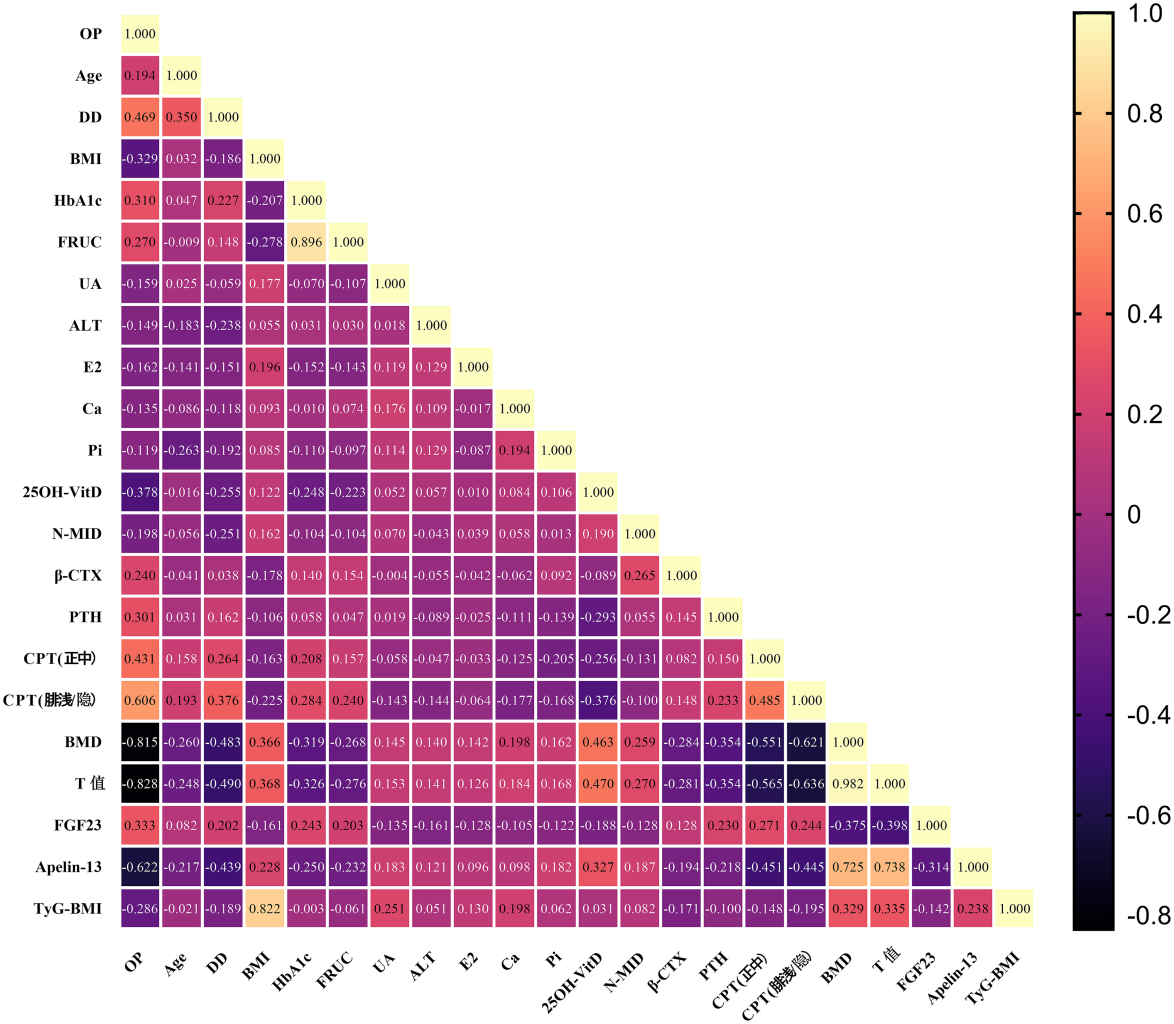
Spearman correlation heatmap of significant clinical indicators among two groups.

Given that OP was not normally distributed, Spearman correlation analysis was performed to assess its univariate correlations with other clinical indicators that showed statistically significant differences between the two groups. As shown in Table 6 and Figure 3, OP was significantly negatively correlated with BMI, UA, ALT, E2, Ca, 25OH-VitD, N-MID, BMD, T-score, Apelin-13, and TyG-BMI (all *p* < 0.05). It was significantly positively correlated with Age, DD, HbA1c, FINS, *β*-CTX, PTH, CPT (median nerve), CPT (superficial peroneal/saphenous nerve), and FGF23 (all *p* < 0.05).

**Table 6:** Spearman correlation analysis between OP and other clinical indicators.

Variables	OP	
	ρ	*p*
Age	0.194**	**0.003**
DD	0.469**	**<0.001**
BMI	−0.329**	**<0.001**
HbA1c	0.310**	**<0.001**
FRUC	0.270**	**<0.001**
UA	−0.159*	**0.014**
ALT	−0.149*	**0.021**
E2	−0.162*	**0.012**
Ca	−0.135*	**0.037**
25OH-VitD	−0.378**	**<0.001**
N-MID	−0.198**	**0.002**
β-CTX	0.240**	**<0.001**
PTH	0.301**	**<0.001**
CPT (median nerve)	0.431**	**<0.001**
CPT (sural/saphenous nerve)	0.606**	**<0.001**
BMD	−0.815**	**<0.001**
T-score	−0.828**	**<0.001**
FGF23	0.333**	**<0.001**
Apelin-13	−0.622**	**<0.001**
TyG-BMI	−0.286**	**<0.001**

*p < 0.05, **p < 0.01, ***p < 0.001; The meaning of the bold values is *P* < 0.05.

**Figure 4:**
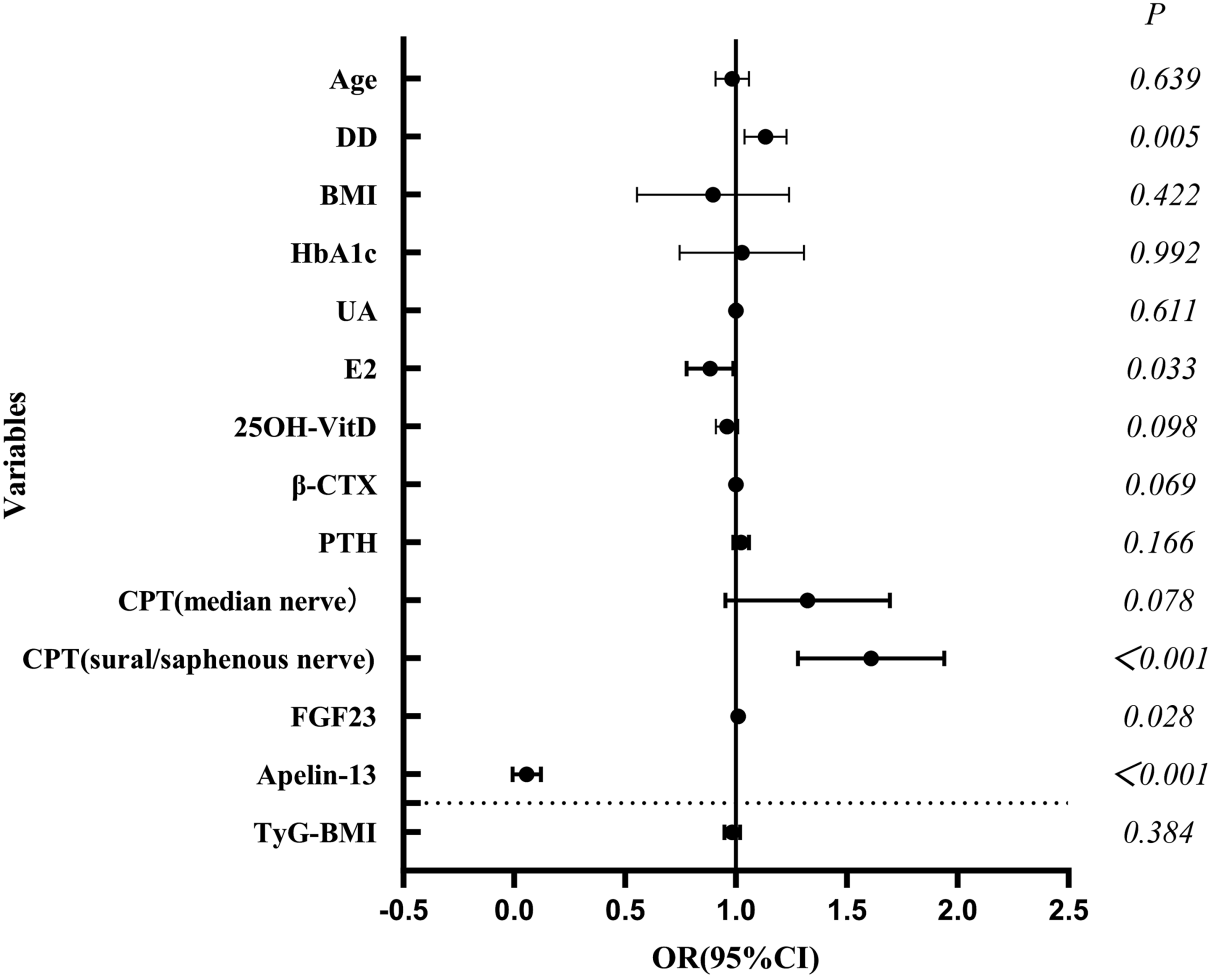
Forest plot of factors influencing OP (logistic regression analysis).

**Figure 5:**
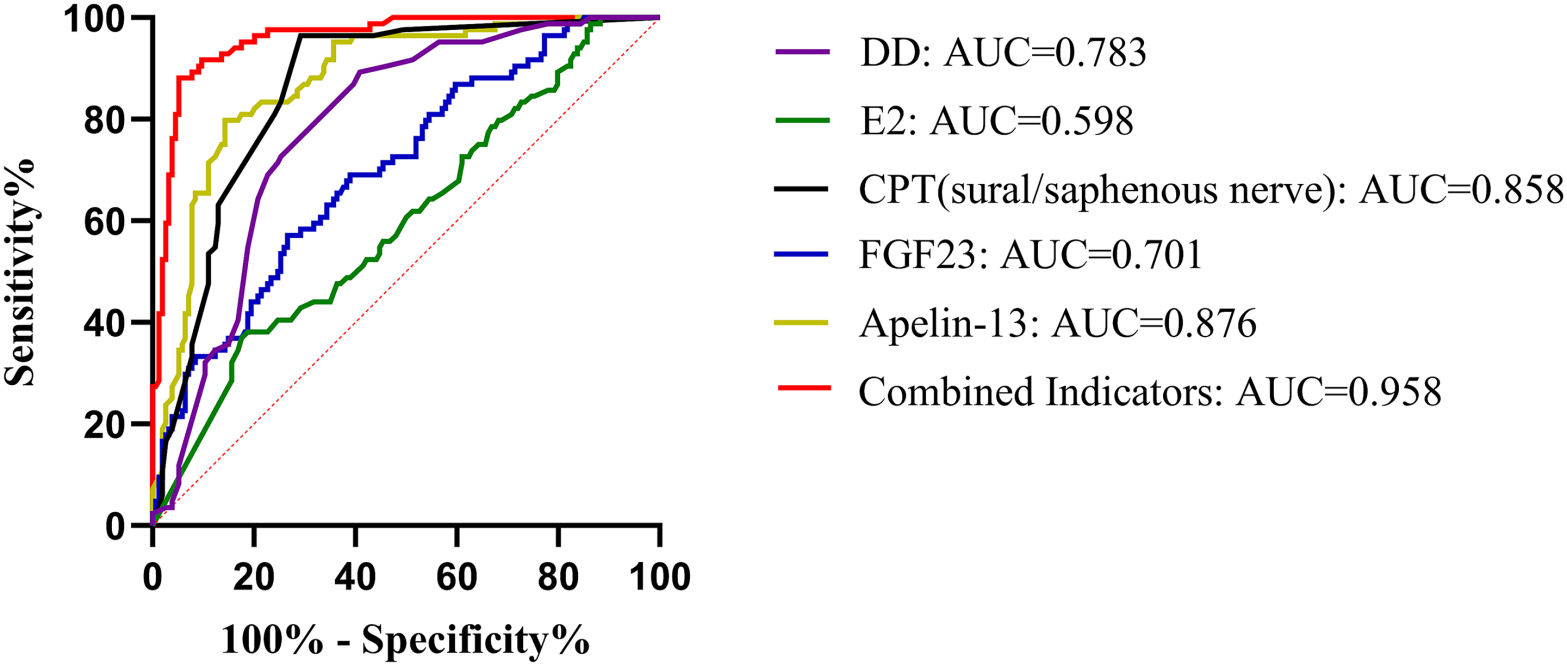
ROC curves of individual and combined indicators for predicting OP.

Logistic regression analysis (Table 7; Figure 4) identified longer DD (OR = 1.13), elevated superficial peroneal/saphenous nerve CPT score (OR = 1.59), and higher FGF23 level (OR = 1.01) as independent risk factors for OP. Higher E2 (OR = 0.88) and Apelin-13 (OR = 0.03) levels were independent protective factors. ROC curves were plotted with DD, E2, CPT (superficial peroneal/saphenous nerve), FGF23, and Apelin-13 (Table 8; Figure 5). The predicted probability from multivariable logistic regression using these five indicators as a combined predictor excellently predicted OP (AUC = 0.958).

**Table 7:** Logistic regression analysis of osteoporosis in postmenopausal patients with T2DM.

Variables	Univariate					Multivariate				
	*β*	S. E	*Z*	*p*	OR (95%CI)	*β*	S. E	*Z*	*p*	OR (95%CI)
Age	0.05	0.02	3.02	**0.003**	1.05 (1.02 ~ 1.08)	−0.02	0.04	−0.47	0.639	0.98 (0.91 ~ 1.06)
DD	0.13	0.02	6.03	**<0.001**	1.14 (1.09 ~ 1.19)	0.12	0.04	2.80	**0.005**	1.13 (1.04 ~ 1.23)
BMI	−0.25	0.05	−4.73	**<0.001**	0.78 (0.70 ~ 0.86)	−0.16	0.20	−0.80	0.422	0.85 (0.58 ~ 1.26)
HbA1c	0.31	0.07	4.61	**<0.001**	1.36 (1.19 ~ 1.55)	0.00	0.14	0.01	0.992	1.00 (0.76 ~ 1.32)
UA	−0.01	0.00	−2.15	**0.032**	0.99 (0.99 ~ 0.99)	−0.00	0.00	−0.51	0.611	1.00 (0.99 ~ 1.01)
ALT	−0.02	0.01	−1.93	0.054	0.98 (0.95 ~ 1.00)					
E2	−0.07	0.03	−2.24	**0.025**	0.93 (0.87 ~ 0.99)	−0.13	0.06	−2.14	**0.033**	0.88 (0.78 ~ 0.99)
Ca	−2.31	1.34	−1.72	0.085	0.10 (0.01 ~ 1.37)					
25OH-VitD	−0.07	0.01	−5.28	**<0.001**	0.93 (0.91 ~ 0.96)	−0.04	0.03	−1.66	0.098	0.96 (0.91 ~ 1.01)
N-MID	−0.03	0.03	−1.03	0.302	0.97 (0.91 ~ 1.03)					
β-CTX	0.01	0.00	2.88	**0.004**	1.01 (1.01 ~ 1.01)	0.00	0.00	1.82	0.069	1.00 (1.00 ~ 1.00)
PTH	0.04	0.01	4.04	**<0.001**	1.04 (1.02 ~ 1.06)	0.02	0.02	1.39	0.166	1.02 (0.99 ~ 1.06)
CPT (median nerve)	0.52	0.10	5.22	**<0.001**	1.69 (1.39 ~ 2.06)	0.25	0.14	1.76	0.078	1.29 (0.97 ~ 1.71)
CPT (sural/saphenous nerve)	0.52	0.07	7.05	**<0.001**	1.67 (1.45 ~ 1.93)	0.46	0.10	4.43	**<0.001**	1.59 (1.29 ~ 1.95)
T-score	−373.21	33213.49	−0.01	0.991	0.00 (0.00 ~ Inf)					
FGF23	0.01	0.00	5.01	**<0.001**	1.01 (1.01 ~ 1.01)	0.01	0.00	2.20	**0.028**	1.01 (1.01 ~ 1.01)
Apelin-13	−4.18	0.58	−7.25	**<0.001**	0.02 (0.00 ~ 0.05)	−3.66	0.82	−4.47	**<0.001**	0.03 (0.01 ~ 0.13)
TyG-BMI	−0.02	0.00	−4.43	**<0.001**	0.98 (0.97 ~ 0.99)	−0.02	0.02	−0.87	0.384	0.98 (0.95 ~ 1.02)

OR, odds ratio. The omnibus test of model coefficients was significant (*p* < 0.001). The Variance Inflation Factor (VIF) for all independent variables was less than 5. The Hosmer-Lemeshow test showed no significance (*p* = 0.193), indicating a good model fit; The meaning of the bold values is *P* < 0.05.

**Table 8:** ROC curve analysis results for individual and combined indicators in predicting OP.

Variables	AUC (95%CI)	Sensitivity	Specificity	Youden’s Index	Cut off	*p*
DD	0.783 (0.724–0.841)	0.893	0.591	0.484	8.500	**<0.001**
E2	0.598 (0.523–0.672)	0.369	0.825	0.194	10.350	**<0.001**
CPT (sural/saphenous nerve)	0.858 (0.811–0.906)	0.964	0.708	0.672	6.000	**<0.001**
FGF23	0.701 (0.633–0.770)	0.571	0.734	0.305	455.163	**<0.001**
Apelin-13	0.876 (0.830–0.921)	0.798	0.857	0.655	1.373	**<0.001**
Combined Indicators	0.958 (0.934–0.982)	0.881	0.948	0.829	0.574	**<0.001**

AUC (95% CI): Area under the receiver operating characteristic curve (and its 95% confidence interval); Sensitivity: Sensitivity/True Positive Rate; Specificity: Specificity/True Negative Rate; Youden’s Index: Sensitivity + Specificity - 1; Cut-off: Cut-off value; The meaning of the bold values is *P* < 0.05.

To assess the independent predictive value of key variables for OP, hierarchical multivariable logistic regression was performed (Table 9). After comprehensive adjustment, longer DD and elevated superficial peroneal/saphenous nerve CPT score remained robust independent risk factors. The independent protective effect of E2 became prominent after adjusting for nerve function and novel biomarkers. The novel biomarkers FGF23 and Apelin-13 demonstrated significant independent risk and protective effects, respectively.

**Table 9:** Hierarchical multivariable logistic regression analysis for osteoporosis in postmenopausal women with T2DM.

Variables	Model 1		Model 2		Model 3		Model 4		Model 5	
	OR (95%CI)	*p*	OR (95%CI)	*p*	OR (95%CI)	*p*	OR (95%CI)	*p*	OR (95%CI)	*p*
Age	1.05 (1.02–1.08)	**0.003**	1.02 (0.98–1.06)	0.290	1.02 (0.97–1.06)	0.493	1.02 (0.97–1.09)	0.423	0.98 (0.91–1.06)	0.639
DD	1.14 (1.09–1.19)	**<0.001**	1.12 (1.07–1.18)	**<0.001**	1.12 (1.06–1.18)	**<0.001**	1.12 (1.05–1.20)	**0.001**	1.13 (1.04–1.23)	**0.005**
BMI	0.78 (0.70–0.86)	**<0.001**	0.91 (0.73–1.14)	0.403	0.94 (0.73–1.21)	0.636	1.02 (0.75–1.38)	0.918	0.85 (0.58–1.26)	0.422
HbA1c	1.36 (1.19–1.55)	**<0.001**	1.32 (1.11–1.56)	**0.001**	1.22 (1.01–1.47)	**0.039**	1.13 (0.90–1.44)	0.297	1.00 (0.76–1.32)	0.992
UA	0.99 (0.99–0.99)	**0.032**	1.00 (0.99–1.00)	0.292	1.00 (0.99–1.00)	0.114	1.00 (0.99–1.00)	0.314	1.00 (0.99–1.01)	0.611
E2	0.93 (0.87–0.99)	**0.025**	0.97 (0.92–1.03)	0.284	0.94 (0.87–1.02)	0.142	0.90 (0.81–0.99)	**0.043**	0.88 (0.78–0.99)	**0.033**
25OH-VitD	0.93 (0.91–0.96)	**<0.001**	0.93 (0.91–0.96)	**<0.001**	0.93 (0.91–0.96)	**<0.001**	0.95 (0.91–0.99)	**0.027**	0.96 (0.91–1.01)	0.098
β-CTX	1.01 (1.01–1.01)	**0.004**	1.01 (1.01–1.01)	**0.023**	1.01 (1.01–1.01)	**0.026**	1.01 (1.01–1.01)	**0.038**	1.00 (1.00–1.00)	0.069
PTH	1.04 (1.02–1.06)	**<0.001**	1.04 (1.02–1.06)	**<0.001**	1.03 (1.01–1.06)	**0.005**	1.02 (0.99–1.05)	0.132	1.02 (0.99–1.06)	0.166
CPT (median nerve)	1.69 (1.39–2.06)	**<0.001**	1.64 (1.31–2.06)	**<0.001**	1.61 (1.27–2.04)	**<0.001**	1.41 (1.11–1.80)	**0.005**	1.29 (0.97–1.71)	0.078
CPT (sural/saphenous nerve)	1.67 (1.45–1.93)	**<0.001**	1.64 (1.39–1.93)	**<0.001**	1.57 (1.32–1.86)	**<0.001**	1.47 (1.23–1.75)	**<0.001**	1.59 (1.29–1.95)	**<0.001**
FGF23	1.01 (1.01–1.01)	**<0.001**	1.01 (1.01–1.01)	**<0.001**	1.01 (1.01–1.01)	**0.019**	1.01 (1.01–1.01)	**0.024**	1.01 (1.01–1.01)	**0.028**
Apelin-13	0.02 (0.00–0.05)	**<0.001**	0.02 (0.01–0.08)	**<0.001**	0.03 (0.01–0.12)	**<0.001**	0.03 (0.01–0.13)	**<0.001**	0.03 (0.01–0.13)	**<0.001**
TyG-BMI	0.98 (0.97–0.99)	**<0.001**	0.98 (0.96–1.01)	0.155	0.98 (0.96–1.01)	0.098	0.98 (0.95–1.00)	0.082	0.98 (0.95–1.02)	0.384

OR, odds ratio, CI, confidence interval. Model 1: Univariate analysis results, unadjusted. Model 2: Model 1 + Adjustment for Age, DD, BMI, HbA1c, UA, TyG-BMI. Model 3: Model 2 + Adjustment for E2, 25OH-VitD, β-CTX, PTH. Model 4: Model 3 + Adjustment for CPT (median nerve), CPT (superficial peroneal/saphenous nerve). Model 5: Model 4 + Adjustment for FGF23, Apelin-13; The meaning of the bold values is *P* < 0.05.

### 3.4 Metabolic characteristics of postmenopausal OP patients with comorbid T2DM

As shown in Table 1, compared to the Non-T2DM (*n* = 82) group, the T2DM with OP (*n* = 84) group exhibited worse glucose metabolism and insulin resistance, altered bone metabolism, a more active inflammatory state, poorer nutritional status, thyroid and pituitary hormone metabolic disorders, as well as higher FGF23 and lower Apelin-13 levels (all *p* < 0.05).

### 3.5 Factors associated with peripheral neuropathy in postmenopausal patients with T2DM

As shown in Table 10, compared to the Non-DPN (*n* = 58) group, the DPN (*n* = 180) group had significantly higher levels of Age, DD, HbA1c, FRUC, *β*-CTX, PTH and FGF23, and lower levels of BMI, 25OH-VitD, N-MID, BMD, T-score, Apelin-13, and TyG-BMI (all *p* < 0.05).

**Table 10:** Comparison of clinical indicators between T2DM patients with and without DPN.

Variables	Total (*n* = 238)	0 (*n* = 58)	1 (*n* = 180)	Statistic	*p*
TC (mmol/L)	4.62 ± 1.13	4.52 ± 1.24	4.65 ± 1.10	*t* = −0.715	0.476
LDL-C (mmol/L)	2.68 ± 0.97	2.45 ± 0.95	2.76 ± 0.97	*t* = −2.117	**0.035**
TP (g/L)	70.46 ± 5.85	72.00 ± 6.28	69.97 ± 5.64	*t* = 2.317	**0.021**
RBC (10^12^/L)	4.54 ± 0.51	4.55 ± 0.52	4.54 ± 0.51	*t* = 0.159	0.874
T4 (ng/mL)	74.48 ± 15.67	73.81 ± 15.69	74.69 ± 15.70	*t* = −0.375	0.708
Ca (mmol/L)	2.32 ± 0.10	2.34 ± 0.09	2.31 ± 0.11	*t* = 1.576	0.116
Pi (mmol/L)	1.17 ± 0.18	1.23 ± 0.17	1.15 ± 0.18	*t* = 2.787	**0.006**
Age (year)	62.00 (56.00, 69.75)	57.00 (53.00, 66.00)	62.00 (57.75, 70.00)	*Z* = −2.598	**0.009**
DD (year)	10.00 (4.00, 15.75)	6.00 (2.00, 10.00)	10.50 (6.00, 17.00)	*Z* = −4.589	**<0.001**
BMI (kg/m^2^)	23.81 (21.93, 26.17)	24.98 (22.60, 27.72)	23.59 (21.77, 25.43)	*Z* = −2.793	**0.005**
HbA1c (%)	8.20 (6.80, 10.20)	7.15 (6.62, 8.28)	8.75 (7.10, 10.80)	*Z* = −4.515	**<0.001**
FRUC (mmol/L)	328.20 (277.57, 390.27)	302.70 (269.05, 336.15)	345.65 (281.53, 415.40)	*Z* = −3.756	**<0.001**
FBG (mmol/L)	6.26 (5.04, 8.30)	6.09 (4.88, 7.62)	6.34 (5.04, 8.38)	*Z* = −1.071	0.284
FCP (ng/mL)	1.38 (0.81, 2.02)	1.51 (0.90, 2.21)	1.29 (0.74, 1.98)	*Z* = −1.361	0.174
FINS (mU/L)	13.08 (9.02, 20.14)	13.33 (8.74, 21.16)	12.98 (9.14, 20.07)	*Z* = −0.260	0.795
2hBG (mmol/L)	16.70 (13.06, 20.82)	15.99 (12.14, 19.81)	16.82 (13.34, 21.08)	*Z* = −1.289	0.197
2hCP (ng/mL)	3.60 (2.30, 5.57)	3.96 (2.57, 5.70)	3.47 (2.19, 5.48)	*Z* = −1.230	0.219
2hINS (mU/L)	29.86 (20.32, 44.61)	32.09 (22.58, 43.79)	29.54 (19.33, 44.87)	*Z* = −1.336	0.182
BUN (mmol/L)	5.36 (4.20, 6.94)	5.60 (4.21, 6.72)	5.30 (4.21, 7.00)	*Z* = −0.389	0.697
Cr (umol/L)	62.55 (55.00, 71.70)	63.95 (56.78, 74.17)	62.40 (54.60, 70.93)	*Z* = −1.166	0.244
UA (umol/L)	297.70 (253.05, 354.73)	299.40 (259.72, 366.85)	296.50 (252.07, 352.30)	*Z* = −1.012	0.312
TG (mmol/L)	1.48 (1.06, 2.26)	1.50 (1.07, 2.26)	1.48 (1.06, 2.25)	*Z* = −0.594	0.552
HDL-C (mmol/L)	1.12 (1.00, 1.33)	1.12 (0.92, 1.34)	1.13 (1.01, 1.33)	*Z* = −0.447	0.655
ALB (g/L)	41.30 (39.60, 43.50)	42.50 (40.42, 44.25)	41.10 (39.38, 43.30)	*Z* = −2.299	**0.022**
AST (IU/L)	21.15 (17.60, 26.03)	21.55 (17.83, 26.40)	21.00 (17.28, 25.72)	*Z* = −0.707	0.479
ALT (IU/L)	16.30 (10.43, 24.00)	17.20 (12.80, 24.00)	15.60 (10.15, 24.00)	*Z* = −1.238	0.216
WBC (10^9^/L)	5.69 (4.91, 6.77)	5.77 (4.83, 6.88)	5.64 (4.92, 6.74)	*Z* = −0.288	0.773
N (10^9^/L)	3.26 (2.44, 4.01)	3.20 (2.37, 4.10)	3.30 (2.45, 3.92)	*Z* = −0.015	0.988
L (10^9^/L)	2.03 (1.64, 2.40)	2.06 (1.71, 2.41)	2.00 (1.62, 2.38)	*Z* = −0.649	0.516
M (10^9^/L)	0.37 (0.30, 0.46)	0.37 (0.30, 0.47)	0.37 (0.30, 0.46)	*Z* = −0.262	0.793
Hb (g/L)	137.50 (128.00, 145.75)	139.50 (128.00, 146.75)	136.00 (128.00, 145.00)	*Z* = −0.794	0.427
T3 (ng/mL)	1.03 (0.89, 1.19)	0.96 (0.86, 1.11)	1.05 (0.91, 1.22)	*Z* = −2.239	**0.025**
FT3 (pg/mL)	2.66 (2.35, 2.93)	2.62 (2.42, 2.88)	2.67 (2.35, 2.94)	*Z* = −0.571	0.568
FT4 (pg/mL)	12.11 (11.15, 13.33)	12.13 (11.15, 13.40)	12.11 (11.16, 13.31)	*Z* = −0.015	0.988
TSH (uIU/mL)	2.95 (1.85, 4.23)	2.78 (1.82, 3.97)	2.96 (1.85, 4.25)	*Z* = −0.534	0.593
Tg (ng/mL)	6.52 (3.59, 14.24)	6.70 (3.80, 11.05)	6.30 (3.60, 15.57)	*Z* = −0.580	0.562
TMAb (IU/mL)	1.69 (0.67, 9.33)	1.92 (0.82, 5.53)	1.65 (0.62, 11.12)	*Z* = −0.004	0.997
TgAb (IU/mL)	8.04 (3.07, 91.76)	8.11 (4.04, 42.25)	7.75 (2.96, 95.96)	*Z* = −0.027	0.978
rT3 (ng/mL)	0.48 (0.40, 0.54)	0.48 (0.40, 0.53)	0.48 (0.40, 0.55)	*Z* = −0.298	0.765
TRAb (IU/L)	0.20 (0.20, 0.20)	0.20 (0.20, 0.24)	0.20 (0.20, 0.20)	*Z* = −0.976	0.329
GH (ng/mL)	0.17 (0.08, 0.56)	0.16 (0.06, 0.34)	0.18 (0.09, 0.56)	*Z* = −1.215	0.224
PRL (ng/mL)	16.21 (12.52, 20.68)	16.34 (12.89, 19.72)	16.16 (12.48, 21.17)	*Z* = −0.245	0.807
FSH (mIU/mL)	51.40 (37.35, 63.88)	46.65 (31.13, 61.80)	52.61 (41.17, 65.67)	*Z* = −2.131	**0.033**
LH (mIU/mL)	18.65 (13.60, 24.75)	16.30 (11.60, 21.67)	19.25 (13.97, 25.55)	*Z* = −2.616	**0.009**
E2 (pg/mL)	12.65 (10.40, 15.40)	13.00 (10.67, 15.88)	12.50 (10.30, 15.22)	*Z* = −1.059	0.290
P (ng/mL)	0.13 (0.10, 0.22)	0.14 (0.10, 0.25)	0.12 (0.10, 0.21)	*Z* = −0.952	0.341
T (ng/mL)	0.04 (0.04, 0.07)	0.06 (0.04, 0.10)	0.04 (0.04, 0.07)	*Z* = −2.174	**0.030**
25OH-VitD (nmol/L)	44.51 (38.39, 55.28)	54.12 (44.53, 63.80)	42.80 (36.52, 51.56)	*Z* = −4.886	**<0.001**
CT (pg/mL)	0.40 (0.40, 0.58)	0.40 (0.40, 0.67)	0.40 (0.40, 0.57)	*Z* = −0.580	0.562
N-MID (ng/mL)	13.60 (12.10, 16.28)	15.25 (12.35, 17.15)	13.40 (12.10, 15.52)	*Z* = −2.222	**0.026**
β-CTX (pg/mL)	421.00 (310.25, 540.75)	360.00 (260.50, 451.50)	445.50 (339.00, 559.25)	*Z* = −3.359	**<0.001**
ALP (IU/L)	75.85 (63.12, 93.62)	71.55 (57.35, 90.00)	78.15 (64.65, 93.70)	*Z* = −1.621	0.105
PTH (pg/mL)	41.75 (33.00, 52.45)	36.50 (30.57, 42.60)	44.20 (33.40, 55.45)	*Z* = −3.446	**<0.001**
MAGE (mmol/L)	4.85 (3.62, 6.61)	4.34 (3.52, 5.38)	5.10 (3.90, 6.92)	*Z* = −2.569	**0.010**
CV (%)	25.04 (20.50, 31.35)	22.25 (19.79, 26.72)	25.69 (20.71, 32.32)	*Z* = −3.056	**0.002**
MBG (mmol/L)	9.11 (7.58, 10.65)	8.75 (7.62, 10.44)	9.23 (7.61, 10.70)	*Z* = −1.198	0.231
SDBG (mmol/L)	2.45 (1.96, 3.19)	2.26 (1.90, 2.65)	2.58 (1.99, 3.30)	*Z* = −2.307	**0.021**
TIR (%)	68.76 (49.40, 83.86)	73.96 (54.45, 85.05)	67.65 (46.89, 82.47)	*Z* = −1.655	0.098
BMD (g/m^2^)	0.99 (0.87, 1.12)	1.19 (1.11, 1.27)	0.91 (0.83, 1.03)	*Z* = −9.759	**<0.001**
T-score	−1.50 (−2.60, −0.50)	0.10 (−0.60, 0.78)	−2.25 (−2.70, −1.20)	*Z* = −9.757	**<0.001**
FGF23 (pg/mL)	426.63 (361.48, 486.17)	387.05 (309.30, 452.35)	435.34 (372.48, 493.14)	*Z* = −3.435	**<0.001**
Apelin-13 (ng/mL)	1.53 (1.17, 2.04)	2.05 (1.81, 2.44)	1.38 (1.13, 1.81)	*Z* = −7.471	**<0.001**
TG/HDL-C	1.34 (0.87, 2.20)	1.35 (0.87, 2.31)	1.33 (0.87, 1.97)	*Z* = −0.537	0.591
TyG-BMI	213.48 (192.22, 237.77)	216.35 (197.13, 253.51)	211.82 (190.55, 234.06)	*Z* = −2.195	**0.028**
HOMA-IR	3.92 (2.38, 6.49)	3.75 (2.44, 5.68)	4.07 (2.28, 6.52)	*Z* = −0.513	0.608
MHR	0.33 (0.25, 0.42)	0.33 (0.27, 0.42)	0.33 (0.24, 0.42)	*Z* = −0.545	0.586
Positive or Negative for HP (*n*, %)				χ^2^ = 0.046	0.831
0	112 (47.06)	28 (48.28)	84 (46.67)		
1	126 (52.94)	30 (51.72)	96 (53.33)		

*t*, two-sample *t*-test; Z, Mann–Whitney U test; χ^2^, Chi-square test; The meaning of the bold values is *P* < 0.05.

**Figure 6:**
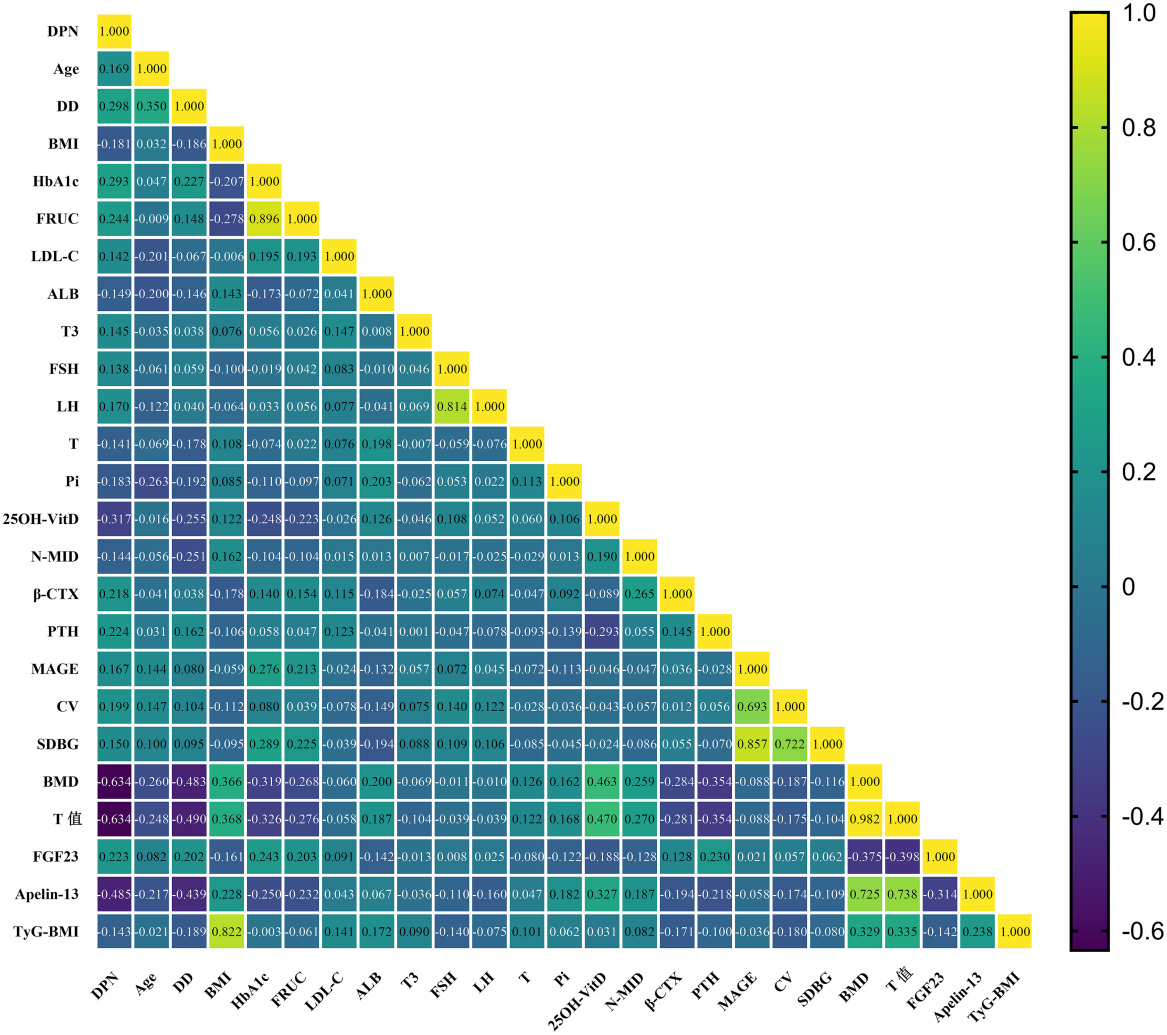
Spearman correlation heatmap of significant clinical indicators among two groups.

Given that DPN was not normally distributed, Spearman correlation analysis was performed to assess its univariate correlations with other clinical indicators that showed statistically significant differences between the two groups. As shown in Table 11 and Figure 6, DPN was significantly negatively correlated with Pi, BMI, ALB, T, 25OH-VitD, N-MID, BMD, T-score, Apelin-13, and TyG-BMI (all *p* < 0.05). It was significantly positively correlated with LDL-C, Age, DD, HbA1c, FRUC, T3, FSH, LH, β-CTX, PTH, MAGE, CV, SDBG, and FGF23 (all *p* < 0.05). No significant correlation was observed with TP.

**Table 11:** Spearman correlation analysis between DPN and other clinical indicators.

**Variables**	**DPN**	
	**ρ**	*p*
LDL-C	0.142*	**0.028**
TP	−0.119	0.066
Pi	−0.183**	**0.005**
Age	0.169**	**0.009**
DD	0.298**	**<0.001**
BMI	−0.181**	**0.005**
HbA1c	0.293**	**<0.001**
FRUC	0.244**	**<0.001**
ALB	−0.149*	**0.021**
T3	0.145*	**0.025**
FSH	0.138*	**0.033**
LH	0.170**	**0.009**
T	−0.141*	**0.029**
25OH-VitD	−0.317**	**<0.001**
N-MID	−0.144*	**0.026**
β-CTX	0.218**	**<0.001**
PTH	0.224**	**<0.001**
MAGE	0.167**	**0.010**
CV	0.199**	**0.002**
SDBG	0.150*	**0.021**
BMD	−0.634**	**<0.001**
T-score	−0.634**	**<0.001**
FGF23	0.223**	**<0.001**
Apelin-13	−0.485**	**<0.001**
TyG-BMI	−0.143*	**0.028**

*p < 0.05, **p < 0.01, ***p < 0.001; The meaning of the bold values is *P* < 0.05.

**Figure 7:**
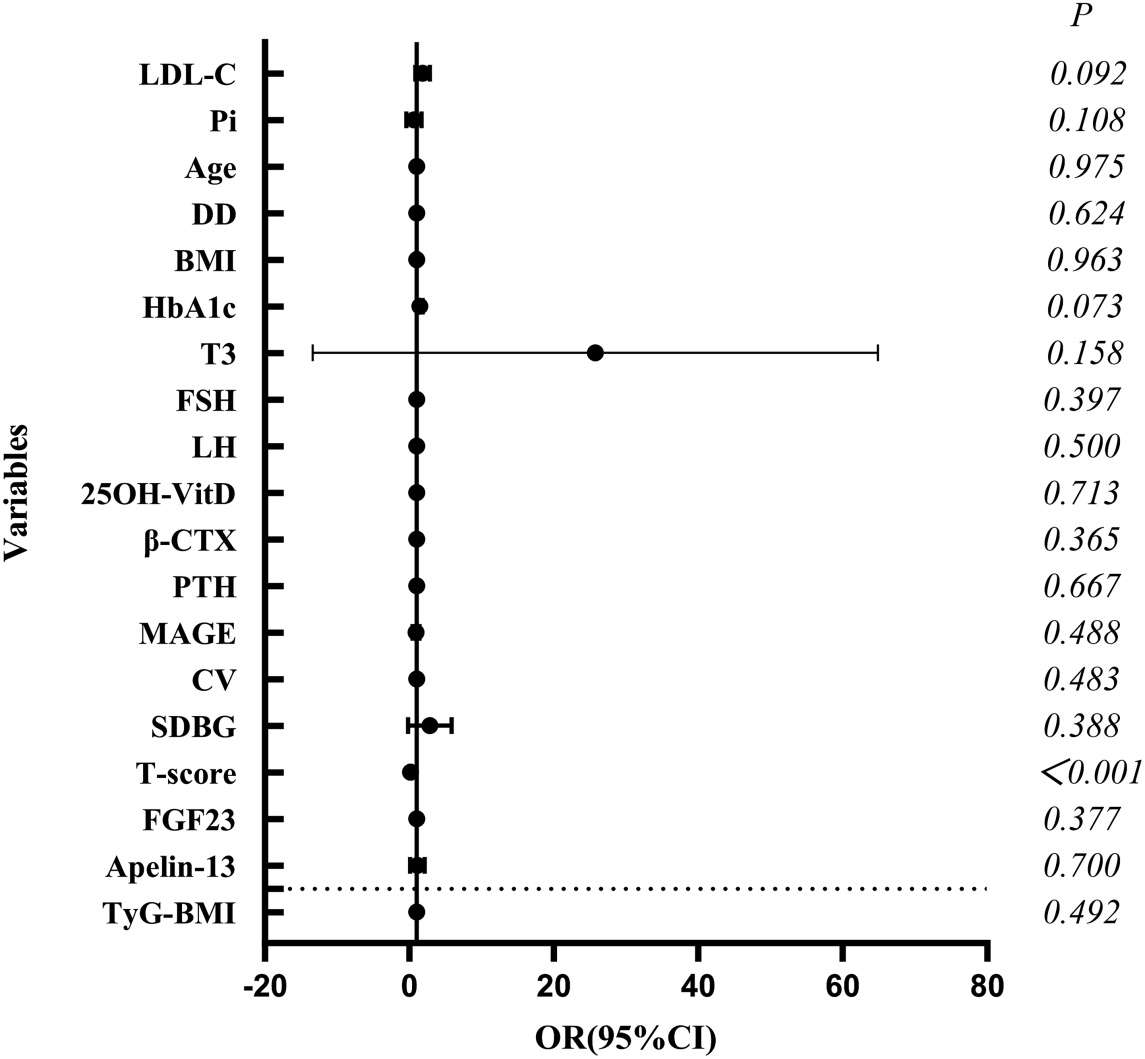
Forest plot of independent factors influencing DPN.

**Figure 8:**
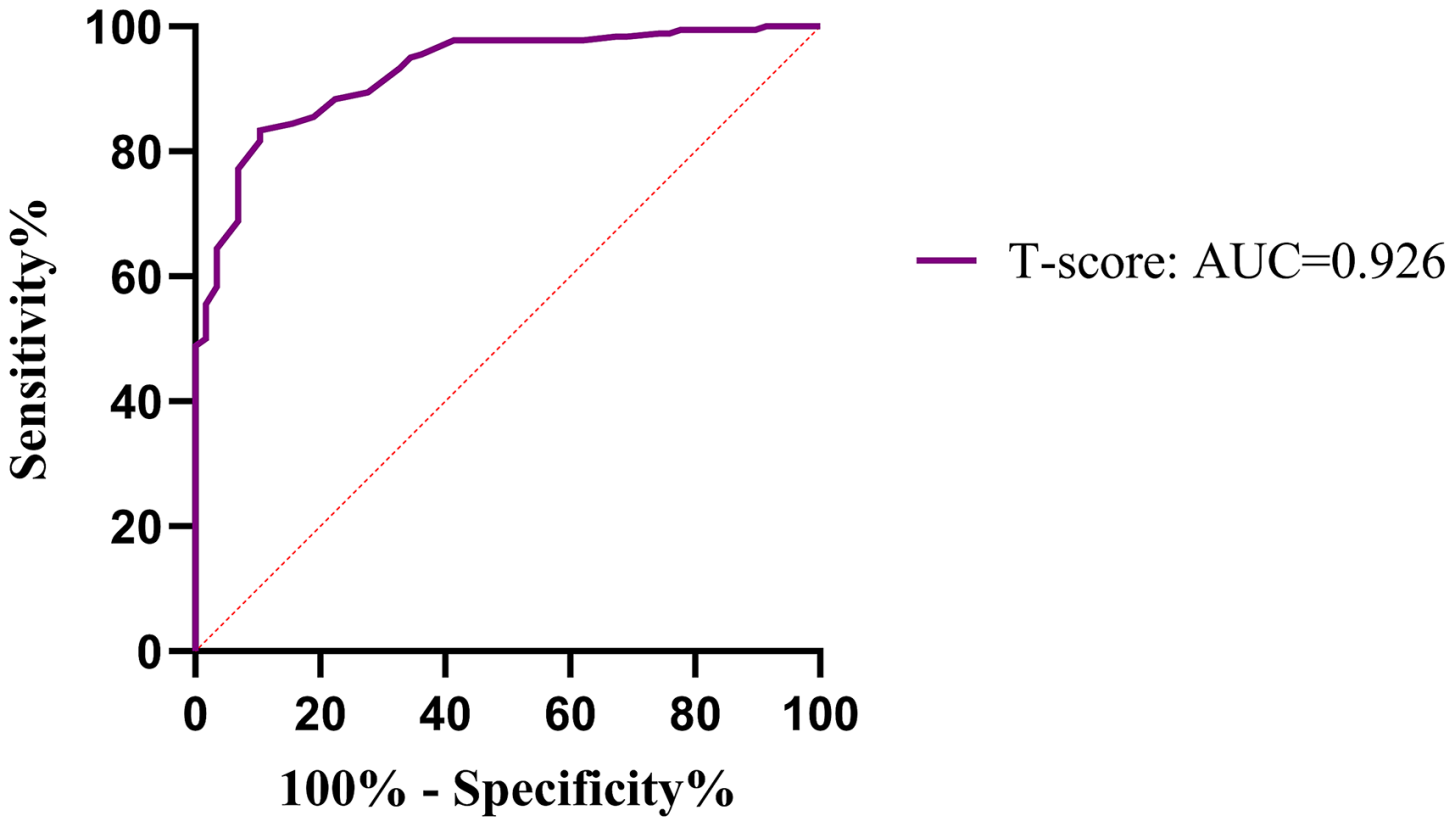
ROC curve of the T-score for predicting DPN.

As shown in Table 12 and Figure 7, multivariate logistic regression identified a lower T-score as the sole independent protective factor against DPN (OR = 0.13, *p* < 0.001), with high predictive value (AUC = 0.926) (Table 13; Figure 8). Associations of FGF23 and Apelin-13 with DPN were not independent after adjusting for bone metabolism markers.

**Table 12:** Logistic regression analysis for DPN in postmenopausal patients with T2DM.

Variables	Univariate					Multivariate				
	β	S. E	*Z*	*p*	OR (95%CI)	β	S. E	*Z*	*p*	OR (95%CI)
LDL-C	0.34	0.16	2.09	**0.037**	1.40 (1.02 ~ 1.92)	0.49	0.29	1.68	0.092	1.64 (0.92 ~ 2.91)
Pi	−2.29	0.85	−2.70	**0.007**	0.10 (0.02 ~ 0.53)	−2.83	1.76	−1.61	0.108	0.06 (0.00 ~ 1.86)
Age	0.05	0.02	2.51	**0.012**	1.05 (1.01 ~ 1.09)	−0.00	0.03	−0.03	0.975	1.00 (0.93 ~ 1.07)
DD	0.10	0.02	4.21	**<0.001**	1.11 (1.06 ~ 1.16)	0.02	0.04	0.49	0.624	1.02 (0.94 ~ 1.11)
BMI	−0.13	0.05	−2.79	**0.005**	0.88 (0.81 ~ 0.96)	0.01	0.15	0.05	0.963	1.01 (0.75 ~ 1.36)
HbA1c	0.42	0.09	4.45	**<0.001**	1.52 (1.26 ~ 1.83)	0.33	0.19	1.79	0.073	1.40 (0.97 ~ 2.01)
ALB	−0.08	0.04	−1.79	0.073	0.93 (0.85 ~ 1.01)					
T3	1.35	0.68	1.98	**0.048**	3.84 (1.01 ~ 14.59)	1.78	1.26	1.41	0.158	5.95 (0.50 ~ 70.79)
FSH	0.02	0.01	2.17	**0.030**	1.02 (1.01 ~ 1.03)	0.02	0.02	0.85	0.397	1.02 (0.98 ~ 1.06)
LH	0.04	0.02	1.97	**0.049**	1.04 (1.01 ~ 1.07)	0.03	0.04	0.67	0.500	1.03 (0.95 ~ 1.11)
T	−5.34	2.82	−1.89	0.058	0.00 (0.00 ~ 1.20)					
25OH-VitD	−0.05	0.01	−4.05	**<0.001**	0.96 (0.93 ~ 0.98)	0.01	0.02	0.37	0.713	1.01 (0.97 ~ 1.05)
N-MID	−0.01	0.02	−0.44	0.663	0.99 (0.96 ~ 1.03)					
β-CTX	0.01	0.00	3.03	**0.002**	1.01 (1.01 ~ 1.01)	0.00	0.00	0.90	0.365	1.00 (1.00 ~ 1.00)
PTH	0.03	0.01	3.07	**0.002**	1.03 (1.01 ~ 1.06)	0.01	0.02	0.43	0.667	1.01 (0.97 ~ 1.05)
MAGE	0.16	0.07	2.19	**0.029**	1.18 (1.02 ~ 1.36)	−0.19	0.28	−0.69	0.488	0.82 (0.48 ~ 1.43)
CV	0.06	0.02	2.70	**0.007**	1.06 (1.02 ~ 1.10)	0.03	0.05	0.70	0.483	1.03 (0.94 ~ 1.13)
SDBG	0.42	0.17	2.44	**0.015**	1.52 (1.09 ~ 2.12)	0.56	0.65	0.86	0.388	1.75 (0.49 ~ 6.22)
T-score	−1.89	0.27	−7.04	**<0.001**	0.15 (0.09 ~ 0.26)	−2.07	0.39	−5.26	**<0.001**	0.13 (0.06 ~ 0.27)
FGF23	0.01	0.00	3.37	**<0.001**	1.01 (1.01 ~ 1.01)	−0.00	0.00	−0.88	0.377	1.00 (0.99 ~ 1.00)
Apelin-13	−1.99	0.32	−6.20	**<0.001**	0.14 (0.07 ~ 0.26)	−0.20	0.51	−0.39	0.700	0.82 (0.30 ~ 2.22)
TyG-BMI	−0.01	0.00	−2.58	**0.010**	0.99 (0.98 ~ 0.99)	0.01	0.01	0.69	0.492	1.01 (0.98 ~ 1.04)

S. E., standard error; OR, odds ratio; CI, confidence interval. The omnibus test of model coefficients was significant (*p* < 0.001), indicating successful model construction. The Variance Inflation Factor (VIF) for all independent variables was less than 5, suggesting no significant multicollinearity. The Hosmer-Lemeshow test showed no significance (*p* = 0.386), indicating a good model fit; The meaning of the bold values is *P* < 0.05.

**Table 13:** ROC curve analysis results of the T-score for predicting DPN.

Variables	AUC (95%CI)	Sensitivity	Specificity	Youden’s Index	Cut off	*p*
T-score	0.926 (0.891–0.961)	0.833	0.897	0.730	−0.950	<0.001

AUC (95% CI), Area under the receiver operating characteristic curve (and its 95% confidence interval); Sensitivity, Sensitivity/True Positive Rate; Specificity, Specificity/True Negative Rate; Youden’s Index, Sensitivity + Specificity - 1; Cut-off, Cut-off value.

To assess the independent predictive value of key variables for DPN, hierarchical multivariable logistic regression was performed (Table 14). After comprehensive adjustment, the BMD T-score consistently showed a strong, independent protective effect across all adjusted models (OR ~0.13–0.15, *p* < 0.001). Notably, associations of traditional risk factors (Age, DD, HbA1c) and novel biomarkers (FGF23, Apelin-13) with DPN lost significance after adjusting for bone metabolism parameters, suggesting their effects may be mediated through bone metabolic pathways.

**Table 14:** Hierarchical multivariable logistic regression analysis for DPN in postmenopausal women with T2DM.

Variables	Model 1		Model 2		Model 3		Model 4		Model 5	
	OR (95%CI)	*p*	OR (95%CI)	*p*	OR (95%CI)	*p*	OR (95%CI)	*p*	OR (95%CI)	*p*
LDL-C	1.40 (1.02–1.92)	**0.037**	1.60 (1.07–2.38)	**0.021**	1.77 (1.04–3.01)	**0.036**	1.63 (0.92–2.88)	0.093	1.64 (0.92–2.91)	0.092
Pi	0.10 (0.02–0.53)	**0.007**	0.18 (0.03–1.18)	0.073	0.17 (0.01–2.92)	0.221	0.07 (0.00–1.96)	0.119	0.06 (0.00–1.86)	0.108
Age	1.05 (1.01–1.09)	**0.012**	1.04 (0.99–1.08)	0.104	1.00 (0.94–1.06)	0.949	1.00 (0.94–1.07)	0.887	1.00 (0.93–1.07)	0.975
DD	1.11 (1.06–1.16)	**<0.001**	1.08 (1.02–1.14)	**0.007**	1.02 (0.95–1.10)	0.538	1.02 (0.94–1.10)	0.637	1.02 (0.94–1.11)	0.624
BMI	0.88 (0.81–0.96)	**0.005**	1.04 (0.84–1.28)	0.718	1.04 (0.80–1.36)	0.772	1.02 (0.76–1.36)	0.917	1.01 (0.75–1.36)	0.963
HbA1c	1.52 (1.26–1.83)	**<0.001**	1.44 (1.18–1.76)	**<0.001**	1.32 (0.97–1.79)	0.075	1.35 (0.95–1.92)	0.096	1.40 (0.97–2.01)	0.073
T3	3.84 (1.01–14.59)	**0.048**	3.35 (0.79–14.23)	0.102	8.11 (0.80–82.38)	0.077	5.47 (0.47–64.07)	0.176	5.95 (0.50–70.79)	0.158
FSH	1.02 (1.01–1.03)	**0.030**	1.01 (1.00–1.03)	0.076	1.03 (1.01–1.06)	**0.009**	1.02 (0.98–1.06)	0.421	1.02 (0.98–1.06)	0.397
LH	1.04 (1.01–1.07)	**0.049**	1.04 (1.01–1.08)	**0.044**	1.07 (1.02–1.12)	**0.011**	1.03 (0.95–1.11)	0.477	1.03 (0.95–1.11)	0.500
25OH-VitD	0.96 (0.93–0.98)	**<0.001**	0.97 (0.94–0.99)	**0.011**	1.01 (0.98–1.05)	0.529	1.01 (0.97–1.05)	0.615	1.01 (0.97–1.05)	0.713
β-CTX	1.01 (1.01–1.01)	**0.002**	1.01 (1.01–1.01)	**0.020**	1.00 (1.00–1.00)	0.259	1.00 (1.00–1.00)	0.317	1.00 (1.00–1.00)	0.365
PTH	1.03 (1.01–1.06)	**0.002**	1.03 (1.01–1.05)	**0.016**	1.00 (0.97–1.04)	0.892	1.01 (0.97–1.04)	0.673	1.01 (0.97–1.05)	0.667
MAGE	1.18 (1.02–1.36)	**0.029**	1.08 (0.91–1.28)	0.399	1.17 (0.93–1.48)	0.184	0.86 (0.51–1.45)	0.567	0.82 (0.48–1.43)	0.488
CV	1.06 (1.02–1.10)	**0.007**	1.05 (1.01–1.10)	**0.033**	1.06 (1.00–1.12)	0.071	1.03 (0.94–1.13)	0.553	1.03 (0.94–1.13)	0.483
SDBG	1.52 (1.09–2.12)	**0.015**	1.24 (0.84–1.85)	0.280	1.71 (0.95–3.08)	0.074	1.60 (0.48–5.40)	0.445	1.75 (0.49–6.22)	0.388
T-score	0.15 (0.09–0.26)	**<0.001**	0.14 (0.08–0.26)	**<0.001**	0.14 (0.08–0.26)	**<0.001**	0.13 (0.06–0.26)	**<0.001**	0.13 (0.06–0.27)	**<0.001**
FGF23	1.01 (1.01–1.01)	**<0.001**	1.00 (1.00–1.01)	0.122	1.00 (0.99–1.00)	0.680	1.00 (0.99–1.00)	0.362	1.00 (0.99–1.00)	0.377
Apelin-13	0.14 (0.07–0.26)	**<0.001**	0.18 (0.09–0.38)	**<0.001**	0.80 (0.32–2.00)	0.630	0.80 (0.29–2.16)	0.654	0.82 (0.30–2.22)	0.700
TyG-BMI	0.99 (0.98–0.99)	**0.010**	0.99 (0.97–1.01)	0.199	1.00 (0.98–1.03)	0.823	1.01 (0.98–1.04)	0.519	1.01 (0.98–1.04)	0.492

OR, odds ratio; CI, confidence interval. Model 1: Univariate analysis results, unadjusted. Model 2: Model 1 + Adjustment for Age, DD, BMI, HbA1c, LDL-C, TyG-BMI. Model 3: Model 2 + Adjustment for Pi, 25OH-VitD, β-CTX, PTH, T-score. Model 4: Model 3 + Adjustment for T3, FSH, LH, MAGE, CV, SDBG. Model 5: Model 4 + Adjustment for FGF23, Apelin-13; The meaning of the bold values is *P* < 0.05.

## 4 Discussion

**Figure 9:**
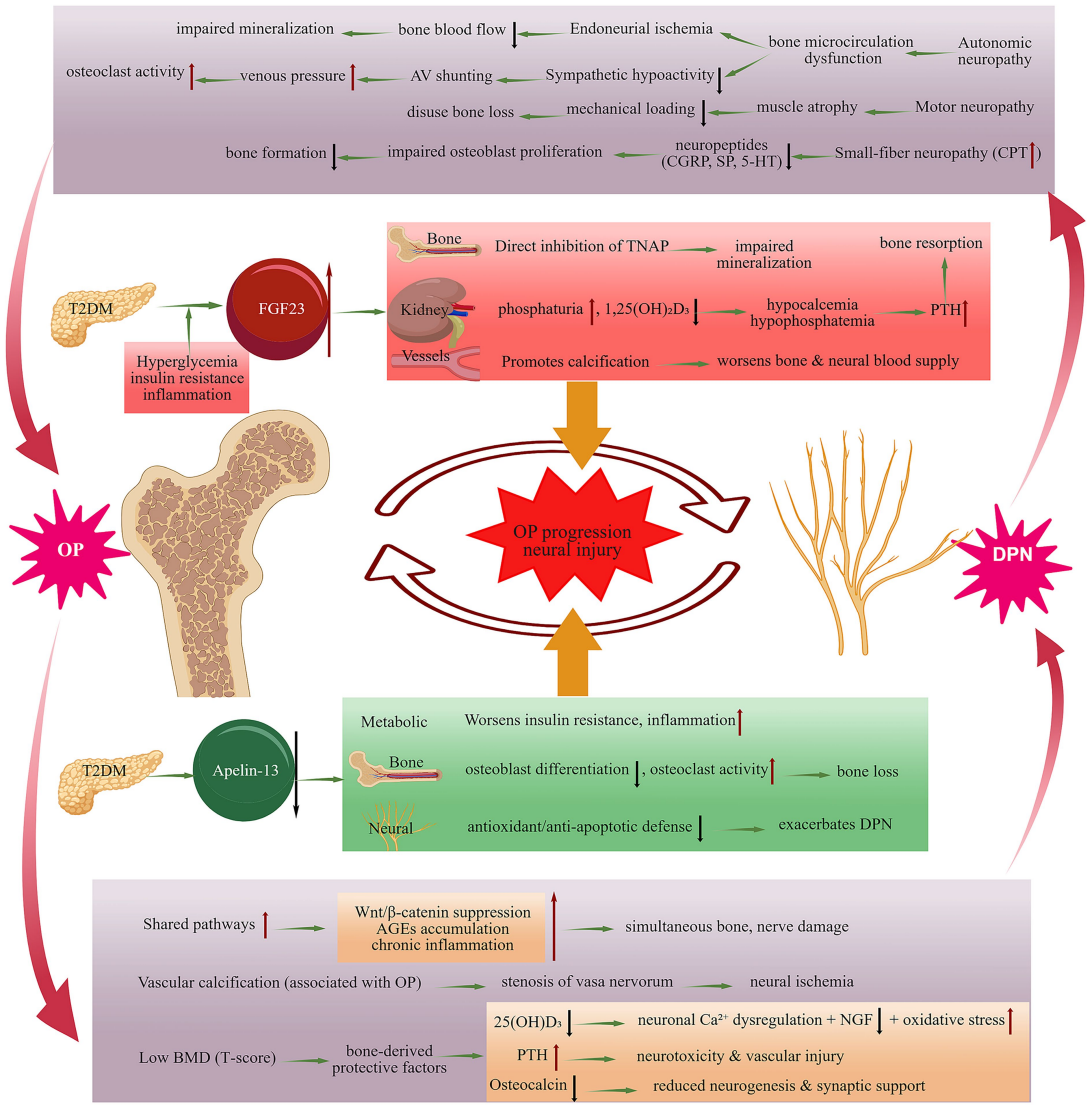
The bidirectional vicious cycle of the “Bone-Neuro Axis” and the critical opposing roles of FGF23 and Apelin-13 in postmenopausal women with T2DM. Red upward arrows indicate an increase, black downward arrows indicate a decrease, and green arrows denote a promoting process.

This cross-sectional study provides preliminary evidence for a close interplay within a ‘bone-neuro axis’ in postmenopausal women with T2DM. Our findings also highlight the opposing associations of FGF23 and Apelin-13 with OP and DPN, suggesting that they may serve as key molecular links within this axis (Figure 9). However, due to the cross-sectional design, these findings are hypothesis-generating and causality cannot be inferred. Our proposed model (Figure 9) is a theoretical framework for future research, not a confirmed causal pathway. Longitudinal prospective studies are urgently needed to test the temporal relationships and causal directions hypothesized in this model.

### 4.1 The bidirectional interaction of the “Bone-Neuro Axis”: a core pathological bridge connecting OP and DPN

The robust associations between nerve dysfunction (elevated CPT score) and OP, and between low BMD and DPN, provide strong clinical evidence for a bidirectional “bone-neuro axis” (23), extending beyond traditional metabolic risk factors. This axis implies a vicious cycle: DPN may drive bone loss by reducing neurotrophic factor secretion (24, 25), causing muscle disuse (26, 27), and disrupting bone microcirculation via autonomic dysfunction (28–30). Conversely, bone density loss, often accompanied by 25-hydroxyvitamin D deficiency, which impairs calcium homeostasis, neurotrophic factor expression, and anti-inflammatory/antioxidant actions, thereby increasing the risk of DPN (31–35). Additionally, vascular calcification often associated with OP may damage nerve nutrient vessels, leading to ischemic nerve injury (32, 36). These findings underscore the need for integrated screening.

### 4.2 FGF23 and Apelin-13: opposing factors associated with bone and nerve health

A key finding of this study is the identification of FGF23 and Apelin-13 as factors independently associated with OP and T-scores, showing opposing directions of association. Based on our findings and previous literature, we hypothesize that FGF23 may be involved in a cycle where metabolic dysregulation stimulates its secretion (37), which then impairs vitamin D metabolism and bone mineralization, accelerates bone resorption and directly inhibits osteoblast activity while promoting inflammation and endothelial dysfunction (38–42). Conversely, the decline in Apelin-13 was associated with lower T-scores and OP. We speculate that Apelin-13 might act via integrated pathways to improve insulin sensitivity and reduce inflammation (43, 44), promote osteogenesis via the Wnt/*β*-catenin pathway and inhibit osteoclastogenesis by suppressing NLRP3 inflammasome activity (45, 46), and provide neuroprotection through anti-inflammatory, antioxidant, and anti-apoptotic actions (19, 47–52). Their imbalance is pivotal to the axis pathology. These mechanistic hypotheses, however, are speculative and require validation in basic and prospective studies. Our cross-sectional data cannot establish whether these associations are causal or the direction of any potential causal relationship. Therefore, the proposed ‘bone-neuro axis’ (Figure 9) should be viewed as a hypothesis-generating framework that requires testing in longitudinal cohort studies and mechanistic experiments.

### 4.3 Clinical utility of a high-performance prediction model

The combined model (DD, E2, CPT score, FGF23, Apelin-13) showed excellent OP prediction (AUC = 0.958), integrating nerve function with novel biomarkers for superior risk stratification. The lack of independent association for many traditional factors (e.g., Age, HbA1c) in our final models suggests their effects may be mediated through pathways closer to end-organ damage, such as by disrupting the FGF23/Apelin-13 axis (53). Our findings support the potential mediating pathway hypothesis: “Hyperglycemia/Disease Duration → Bone Metabolic Disorders (e.g., elevated FGF23, decreased Apelin-13) → Peripheral Neuropathy.”

### 4.4 Distinct phenotype of OP with comorbid T2DM

Compared to OP alone, patients with comorbid T2DM exhibited a more adverse profile: severe glucolipid metabolic disorders and insulin resistance (54), widespread endocrine dysfunction, active chronic inflammation, and a paradoxical high-BMD yet high-bone-turnover state linked to hyperglycemia, insulinemia, AGEs, and mineral axis dysfunction (54–56). Notably, they had higher FGF23 and lower Apelin-13 levels. This defines a “high BMD–high fracture risk” phenotype, necessitating management beyond BMD to include comprehensive assessment of bone turnover, metabolic control, nutrition, and cytokine levels, thus requiring multi-targeted strategies.

### 4.5 Study innovations and limitations

We innovatively focused on a high-risk, homogeneous population to first elucidate the nodal roles of FGF23 and Apelin-13 and build a clinically useful predictive model. Limitations include the cross-sectional design, single-center origin, unaccounted confounders (e.g., medication types), and unexplored the molecular mechanisms of FGF23 and Apelin-13.

## 5 Conclusions and prospects

This study is the first to systematically reveal, in postmenopausal women with T2DM, that decreased BMD is associated with classical bone metabolism disorders, an imbalance of FGF23 and Apelin-13, and impaired nerve function. OP and DPN exhibit shared associated risk features including long DD, high FGF23, and low Apelin-13. A combined model incorporating DD, E2, nerve function scores, FGF23, and Apelin-13 shows potential predictive value for OP risk, and BMD also demonstrates predictive value for DPN. Apelin-13 and FGF23 may be key connecting factors associated with the “bone-neuro axis” and could become potential targets for the synergistic prevention and treatment of both OP and DPN. Comprehensive assessment of bone metabolism, nerve function, and these novel markers may be beneficial for this high-risk population. However, due to the cross-sectional design, causality cannot be established. Therefore, these findings are hypothesis-generating only, and longitudinal studies are essential to validate the observed associations and the proposed ‘bone-neuro axis’ model.

Several methodological limitations should be acknowledged when interpreting our findings. First, the cross-sectional design precludes any inference of causality; the observed associations between FGF23, Apelin-13, and OP/DPN require confirmation in prospective cohort studies. Second, despite our efforts to minimize overfitting by using stepwise selection and limiting the number of variables, the relatively modest sample size, particularly in subgroup analyses (e.g., OP group, *n* = 84), may still predispose to model instability. Specifically, the EPV for the OP prediction model was only 7.0, which is below the commonly recommended threshold of 10. Consequently, the high AUC values reported (e.g., 0.958 for OP) may be optimistically biased due to overfitting. External validation in an independent cohort is imperative to confirm the predictive performance of our models. Selection bias is a major concern. All participants were recruited from a single tertiary referral hospital. This population likely represents a more severe disease spectrum (e.g., longer diabetes duration, poorer glycemic control, higher DPN prevalence) than community-dwelling postmenopausal women with T2DM. Consequently, our findings—including the strength of associations and the AUC of the predictive models—may be overestimated and cannot be directly generalized to the broader community. External validation in independent, multi-center, community-based cohorts is essential. Third, residual confounding from unmeasured variables cannot be entirely excluded. Our study did not collect or adjust for several factors known to affect bone and nerve health, including: (1) specific glucose-lowering medications (e.g., metformin with potential neuroprotective effects; SGLT2 inhibitors or thiazolidinediones that affect bone metabolism); (2) dietary calcium intake and vitamin D supplementation; and (3) lifestyle factors such as physical activity and sunlight exposure. The absence of these variables means that the independent associations we report for FGF23 and Apelin-13 could be partially confounded or mediated by these unmeasured factors. For example, if a neuroprotective medication was more commonly used in the non-DPN group, the true association between a risk factor and DPN would be underestimated. Similarly, higher dietary calcium intake or regular physical activity might independently protect against both OP and DPN, and their omission may have inflated the apparent strength of the associations reported for the novel biomarkers. Future studies must systematically collect and adjust for these potential confounders. Although a history of prior fracture was recorded, we did not include it in our analysis for two reasons. First, accurately distinguishing between osteoporotic (fragility) fractures and traumatic fractures is challenging in retrospective studies, raising concerns about data reliability. Second, our OP definition was based strictly on BMD T-score (≤ − 2.5), and given the expected strong correlation between fragility fracture history and low BMD, including both would introduce collinearity. Nevertheless, this decision precludes evaluation of the independent contribution of fracture history beyond BMD, which remains a limitation. Furthermore, the sample size of the non-diabetic OP control group (*n* = 82) was smaller than that of the T2DM group (*n* = 238). While non-parametric tests were used to mitigate this imbalance, the reduced sample size may still limit the statistical power for detecting true differences in certain comparisons (e.g., Table 1) and increases the risk of type II errors. These findings should be considered preliminary. Finally, both FGF23 and Apelin-13 were measured at a single time point. Although blood samples were collected under standardized conditions (fasting, morning) to minimize diurnal variation, single measurements cannot fully capture intra-individual biological variability. This limitation likely introduces non-differential misclassification, biasing effect estimates toward the null (i.e., underestimating true associations). Thus, the true relationships between these biomarkers and OP/DPN may be stronger than reported. Future prospective studies with repeated measurements are needed to confirm these findings.

Future large-scale prospective cohort studies are needed to validate the predictive value and causal relationships of these markers. Basic experiments are required to deeply elucidate the specific molecular mechanisms of FGF23 and Apelin-13 in the “diabetes-bone-neuro” crosstalk to advance the development of related targeted therapeutic strategies (e.g., exploring APJ receptor agonists, lifestyle, or nutritional interventions to elevate Apelin-13 levels or enhance its signaling; using phosphate binders, vitamin D analogs, or FGF23-neutralizing antibodies to target the FGF23 axis). Future studies should also consider confounding factors such as the types of glucose-lowering medications, diet, exercise habits, and sunlight exposure.

## Data Availability

The raw data supporting the conclusions of this article will be made available by the authors, without undue reservation.
